# Molecular Insights into the Formation and Functionalization of Carbon Nanodots: From Precursor Intermediates to Surface Chemistry Quantification

**DOI:** 10.1002/anie.202515073

**Published:** 2025-08-08

**Authors:** Emanuele Giuliani, Maria Sbacchi, Serena Agostini, Giacomo Filippini, Beatrice Bartolomei, Pierangelo Gobbo, Maurizio Prato

**Affiliations:** ^1^ Department of Chemical and Pharmaceutical Sciences INSTM UdR Trieste University of Trieste via Licio Giorgieri 1 Trieste 34127 Italy; ^2^ Malvern Panalytical Ltd Grovewood Road, Malvern Worcestershire WR14 1XZ UK; ^3^ Center for Cooperative Research in Biomaterials (CIC BiomaGUNE) Basque Research and Technology Alliance (BRTA) Paseo de Miramón 194 Donostia San Sebastián 20014 Spain; ^4^ Basque Fdn Sci, Ikerbasque Bilbao 48013 Spain

**Keywords:** Carbon nanodots, Gel permeation chromatography, Interfacial chemistry, Molecular weight, Purification and characterization

## Abstract

Understanding the mechanistic pathways underlying carbon nanodot (CND) formation is essential for the rational design of synthesis strategies and the development of well‐defined nanomaterials. In this work, we combine chromatographic isolation, spectroscopic characterization, and synthetic validation to identify key molecular intermediates in the early stages of CND formation from arginine (Arg) and ethylenediamine (EDA) under hydrothermal conditions. Using reverse‐phase high‐performance liquid chromatography, ^1^H/^13^C nuclear magnetic resonance (NMR) spectroscopy, and mass spectrometry, we isolate and structurally confirm multiple reaction intermediates, including species arising from Arg cyclization and EDA‐derived condensation products. Each proposed intermediate was validated *via* direct synthesis and comparative analysis. Fully formed CNDs were then isolated and extensively characterized through several techniques. Multi‐detection gel permeation chromatography enabled the determination of absolute number average molecular weight (*M_n_* = 4,400 ± 458 g mol^−1^), dispersity (*Đ* = 1.34), and hydrodynamic diameter (2.8 ± 0.2 nm). Quantification of amine functionalities using Kaiser test and ^19^F NMR, upon condensation with a fluorinated aldehyde probe, established the presence of 6–7 amines per particle. Together, this systematic approach not only elucidates the mechanistic origin of CND formation but also establishes a robust framework for molecular‐level CND design and functionalization.

## Graphical abstract

Key molecular intermediates formed in the early stages of carbon nanodot (CND) formation from arginine and ethylenediamine were identified. The resulting CNDs were isolated and thoroughly characterized by several techniques. Multi‐detection gel permeation chromatography enabled absolute determination of the molecular weight, dispersity, and hydrodynamic diameter, as well as estimation of the number of reactive surface amines per particle.

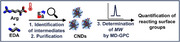

## Introduction

After nearly two decades of intensive research, the formation mechanism of carbon nanodots (CNDs) remains an elusive puzzle. Despite the explosive growth of interest in these tiny luminescent materials, surprisingly little effort has been dedicated to unraveling the complexities of their synthesis.^[^ ^1^, ^2^ ^]^ The transformation of small molecular precursors into nanoscale carbon architectures is still cloaked in ambiguity, with only fragments of the full picture emerging. Cracking this code could not only deepen our fundamental understanding but also unlock smarter and more controlled synthetic strategies.

Recent studies have started to illuminate the formation pathways of these nanoparticles, hinting at the involvement of low‐molecular‐weight intermediates—molecular ghosts that momentarily flicker into existence before vanishing into the final nanoparticle structure. These short‐lived species may hold the key to understanding how molecular fluorophores form within the carbon core, as well as how heteroatoms are doped into the structure.^[^ ^3^, ^4^, ^5^, ^6^, ^7^, ^8^ ^]^ However, in most reported cases, these intermediates have not been isolated, nor has their structure been definitively confirmed through direct synthesis. The core challenge lies in the complicated nature of CND synthesis itself—where a cascade of reactions occurs simultaneously, making it extraordinarily difficult to deconvolute the sequence of events that give rise to the final nanodot.

To address this complexity, the field urgently needs a rigorous and systematic investigation, grounded in the principles of organic chemistry. A sharper mechanistic lens would allow us to demystify the particle formation process and finally establish a rational approach to CND design—one that does not rely on guesswork or serendipity.

In parallel, recent reports have thrown much‐needed light on another long‐overlooked factor, namely purification.^[^ ^9^, ^10^, ^11^, ^12^ ^]^ The removal of molecular byproducts formed during synthesis is anything but trivial. If left behind, these remnants can hijack the properties of the final material, leading to false attributions, and misinterpretation of results. Yet, despite repeated warnings in the literature, purification is often approached too casually—if not outright neglected. This persistent flaw continues to muddy the waters, hindering the field's progress and skewing our understanding of what CNDs truly are and can do.

Moreover, to date, no extensive and detailed investigations have attempted to determine the molecular weight of CNDs. Establishing this fundamental chemical property would pave the way for quantifying surface‐reactive groups and enable the rational design and precise characterization of CND hybrids *via* the conjugation, for example, of small functional molecules, polymers, biomolecules, and even other nanostructures.^[^ ^13^, ^14^, ^15^, ^16^ ^]^ The determination of CND molecular weight and molecular weight distribution would represent a crucial step toward transforming these enigmatic nanostructures into reliable, multifunctional platforms.

The combination of a sound purification protocol, combined with a solid analytical tool able to confirm the purification level, would certainly improve the state‐of‐the‐art in this still growing field.

In this work, we delve into the synthesis and molecular characterization of CNDs derived from *L*‐arginine (Arg) and ethylenediamine (EDA), a system previously developed and optimized by our group.^[^ ^17^ ^]^ Over recent years, these nanoparticles have been thoroughly explored, and the versatility of this synthetic route has been showcased across multiple studies. The protocol accommodates a wide range of doping agents and diamine variations, enabling fine‐tuning of CND properties such as fluorescence behavior, redox activity, and even chiroptical features.^[^ ^18^ ^]^ This adaptability underscores its value as a robust platform for engineering advanced nanomaterials.

Recently, we monitored the in situ evolution of these CNDs using a suite of spectroscopic techniques, capturing a step‐by‐step transformation that unfolds through four critical stages: 1) aggregation of small organic precursors, 2) formation of a dense carbon‐rich core surrounded by a loose shell, 3) collapse of this peripheral shell, and 4) progressive aromatization of the carbon core.^[^ ^19^ ^]^ While this mechanistic framework marks a significant advance, direct chemical insight into the molecular intermediates remained elusive, until now.

In the present study, we isolate and structurally identify the molecular derivatives formed during the initial stages of the hydrothermal process, using a comprehensive combination of analytical and spectroscopic techniques. These findings offer a rare molecular glimpse into the transitional species that bridge the gap between the initial monomers and the final nanoparticle architecture.

To further deepen our understanding of CNDs, after rigorously validating sample purity, we used multi‐detection gel permeation chromatography (MD‐GPC) to determine molecular weight, dispersity, and quantify the density of reactive surface groups. These parameters are critical for predicting and controlling downstream conjugation strategies in hybrid nanomaterials and for developing novel carbon dots‐based nano‐catalytic systems.^[^ ^18^, ^20^ ^]^ By bridging organic chemistry with materials science, this work demonstrates how a molecular‐level understanding can inform a more intelligent, intentional design of the reaction environment. It underscores the crucial—but often underappreciated—role of purification and structural characterization in unlocking rational synthesis strategies for next‐generation carbon‐based nanomaterials.

## Results and Discussion

### Molecular Insights into CND Formation

To enable the rational design of CNDs synthesis, it is essential to unravel the underlying processes governing their formation. A major blind spot in the current synthetic landscape lies in the early‐stage interactions and reactions of the molecular precursors. In this work, we address this knowledge gap by employing a combination of analytical and spectroscopic techniques to probe the initial steps of CND formation.

Specifically: 1) molecular intermediates arising during CND synthesis were isolated using reverse‐phase high‐performance liquid chromatography (HPLC) and subsequently characterized *via* nuclear magnetic resonance (NMR) spectroscopy and mass spectrometry (MS); then 2) the chemical identity of these isolated species was confirmed through direct synthesis of the proposed target compounds. This systematic and rigorous approach is unprecedented in the field and lays the foundation for a general workflow for mechanistic investigations of CND formation.

**Figure 1:**
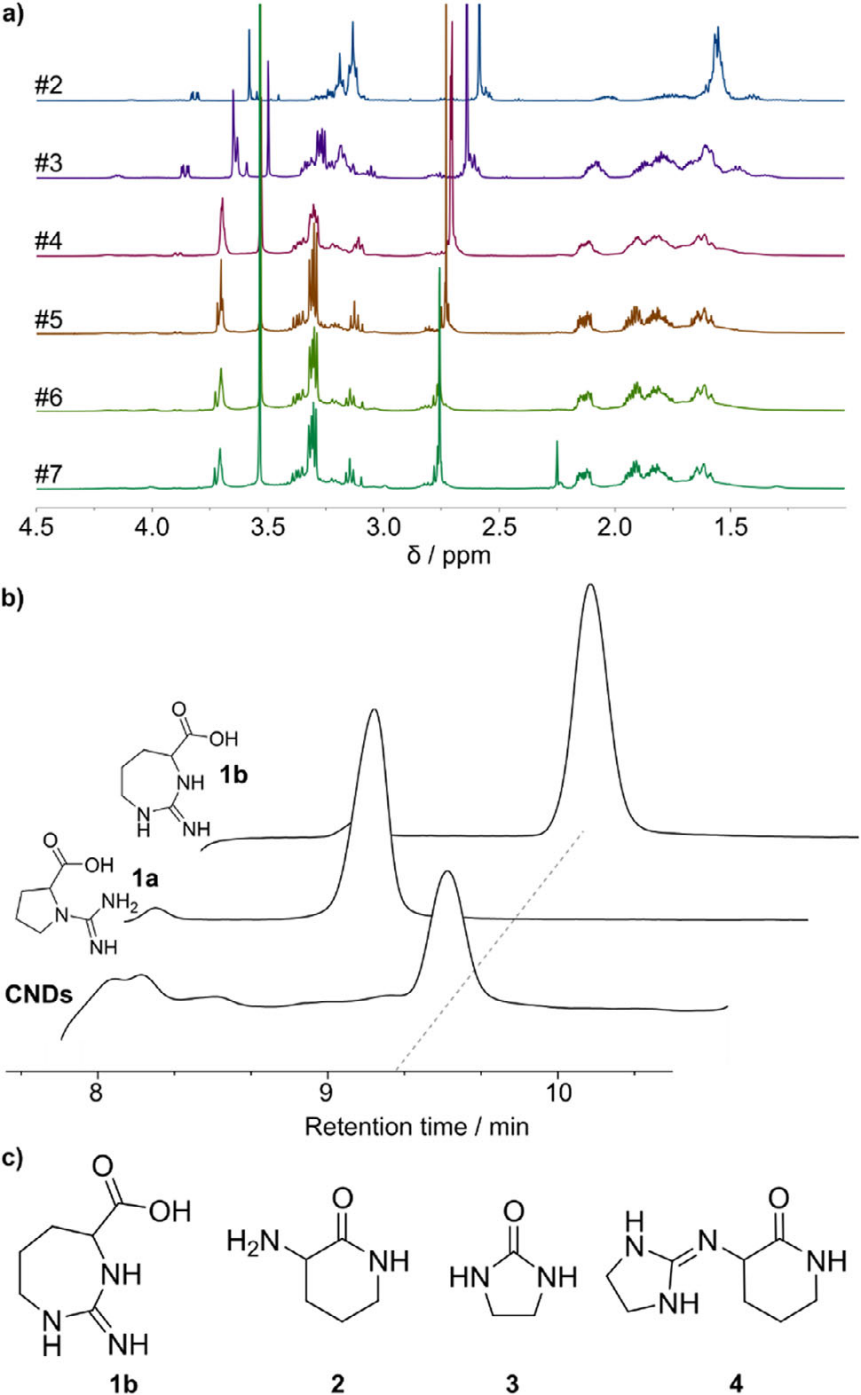
a) ^1^H NMR spectra (D_2_O, 400 MHz) with the region of interest of the crude reaction mixture monitored between #2–7 MW cycles. b) Semipreparative C8‐HPLC traces of CNDs, and for comparison, those of the synthesized compound **1a** and **1b**, monitored at 215 nm. c) Chemical structure of the identified molecular derivatives.

In particular, CNDs were synthesized hydrothermally following a previously reported protocol.^[^ ^17^ ^]^ A mixture of Arg and EDA was subjected to microwave (MW) irradiation using a power cycling regime (15 s on, 5 s off) with a peak temperature of 250 °C and a minimum of 240 °C, for 1 to 12 cycles. After each cycle, the crude mixture was analyzed by ^1^H NMR. Within the 2–7 cycle window, we observed the emergence and subsequent depletion of a molecular species corresponding to a doublet of doublets at 3.7 ppm (Figure 1a).

To identify this intermediate, the compound was isolated *via* reverse‐phase HPLC. The resulting chromatograms revealed multiple peaks, indicating a complex mixture of molecular species (Figure S1). Fractionation of the chromatogram identified a component with a retention time of 9.5 min, which was extensively characterized by 1D and 2D NMR and mass spectrometry, revealing a species with a m/z of 158 (Figures S7‐S11). These data suggested a deamination product of Arg, aligning with previous reports of Arg undergoing cyclization under basic, high‐temperature conditions.^[^ ^21^, ^22^ ^]^ Two candidate structures (compounds **1a** and **1b**, Figure 1b) are in principle possible. Both were synthesized and characterized independently (see Supporting Information), and comparison with the isolated intermediate confirmed compound **1b** as the product formed during CND synthesis (Figure 1b).

Using our HPLC method, we also isolated another early‐stage intermediate with a retention time of 5.9 min and an m/z of 115. Its ^1^H and ^13^C NMR spectra are reported in Figures S3 and S4. Based on literature evidence,^[^ ^23^ ^]^ this compound (**2**, Figure 1c) likely results from hydrolysis of the guanidinium group in Arg, forming ornithine and urea, followed by thermal lactamization. To verify this, compound **2** was synthesized independently (see Supporting Information). Its spectroscopic and chromatographic signatures matched those of the isolated species, confirming its identity. Importantly, these findings support the hypothesis that cyclization reactions are central to the initial stages of CND formation.

Notably, none of the intermediates identified so far involve EDA, a critical co‐precursor in CND synthesis. To probe its role, comparative chromatographic analyses were performed on reaction mixtures synthesized with and without EDA (Figures S1–2). The presence of EDA yielded additional peaks in the 10–12 min retention time range. Fractions from this region revealed two distinct species. The first, with an *m/z* of 87 and a singlet at 3.5 ppm in the ^1^H NMR spectrum, which was consistent with 2‐imidazolidinone, a known condensation product of urea and EDA.^[^ ^24^ ^]^ This was confirmed by synthesizing compound **3** (Figure 1c) *via* MW‐assisted reaction of urea and EDA (see SI). The product matched the isolated intermediate in MS, NMR, and HPLC retention time.

The second species, also isolated from the 10–12 min of retention range, showed a *m/z* of 183. Thus, we hypothesize that this compound (**4**, Figure 1c) forms *via* condensation of compound **2** with compound **3**. To test this, compound **4** was synthesized through a modified literature procedure involving the reaction of 2‐methylthio‐2‐imidazoline hydroiodide with compound **2** under thermal conditions (see Supporting Information).^[^ ^25^ ^]^ HPLC monitoring confirmed the formation of the expected product.

Altogether, our findings demonstrate that cyclic intermediates are key stepping stones in the pathway from small‐molecule precursors to final carbon nanodots. Once these ring structures are established, the reaction cascade progresses toward the formation of fully developed CNDs, probably through an aromatization step to form the core of the nanoparticles. These insights contribute a significant advancement in the mechanistic understanding of CND formation and highlight the value of integrating synthetic chemistry, purification, and detailed structural characterization in nanomaterials research.

### Assessment of CND Purity and Determination of the Molecular Weight

The accurate determination of the CNDs molecular weight and dispersity (*Đ*) is a challenging task and requires a sample of high purity. In this regard, to ensure an effective removal of molecular impurities, including small cyclic molecules involved in their formation pathway, the CNDs were purified *via* filtration and extensive dialysis against Milli–Q water. Sample purity was confirmed *via* both ^1^H NMR spectroscopy and Diffusion‐Ordered NMR Spectroscopy (DOSY) (Figures S52). The ^1^H NMR spectrum displayed broad resonances and the absence of sharp, low‐molecular‐weight signals, indicating the effective removal of unreacted precursors and molecular byproducts.^[^ ^10^ ^]^ DOSY NMR further supported this observation by revealing a single diffusing species with a diffusion coefficient of *D_0_ *= (2.75 ± 0.24) × 10^−10^ m^2^ s^−1^, consistent with literature values for similarly prepared CNDs.^[^ ^26^ ^]^

Further characterization was conducted using atomic force microscopy (AFM), dynamic light scattering (DLS), zeta potential analysis, attenuated total reflectance‐Fourier transform infrared spectroscopy, and UV–vis absorption and fluorescence spectroscopy (see Supporting Information). Together, these results confirmed successful synthesis of Arg/EDA‐derived CNDs and aligned well with previous reports.

To determine the molecular weight distribution of CNDs, we employed MD‐GPC. This technique, which combines refractive index (RI), UV–vis, and light scattering detectors, enables absolute molecular weight determination without requiring calibration standards.^[^ ^27^, ^28^ ^]^ Additionally, a viscometer detector provided insight into hydrodynamic properties.

**Figure 2:**
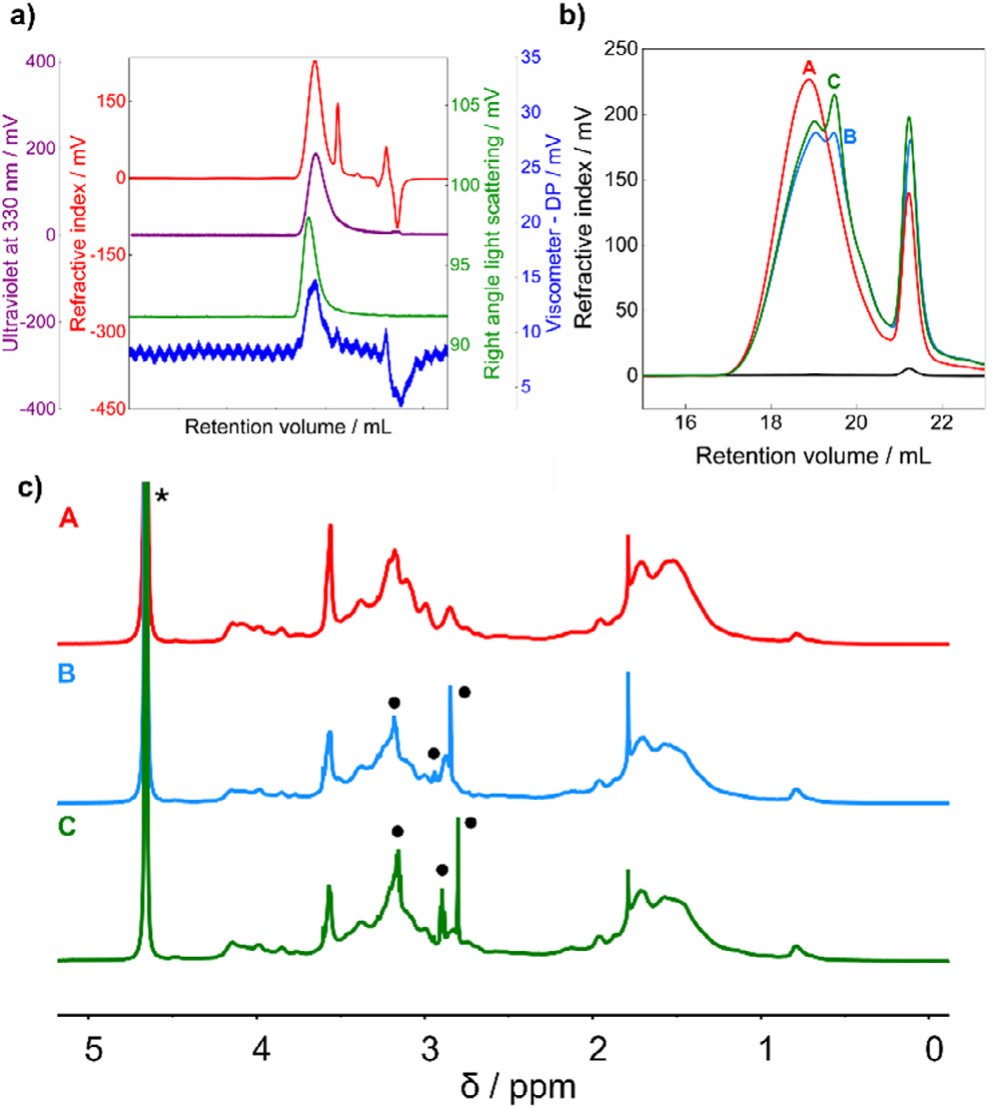
a) Chromatograms of CNDs (batch A, sample concentration = 5 mg mL^−1^, 0.1 M NaNO_3_ + 0.5 v/v% acetic acid (pH 2.6) on cationic columns). From top to bottom: refractive index curve (red), UV curve at 330 nm (purple), light scattering curve (green), and viscometer curve (blue), respectively. b) Refractive index curve overlay of the three different CND batches (5 mg mL^−1^, 0.1 M NaNO_3_ + 0.5 v/v% acetic acid (pH 2.6) on cationic columns) and a blank sample (black curve). Red curve corresponds to batch A, while the light blue and green curves correspond to batch B and C, respectively. c) ^1^H NMR spectra of the same three batches of CND, referenced against deuterium oxide residual peak (*δH *= 4.79 ppm), with the region of interest (4.5–2.5 ppm) showing sharp signals in the light blue and green spectra (batch B and C, respectively).

Given the positive zeta potential of CNDs in 0.1 M sodium nitrate solution containing 0.5% v/v acetic acid (+12.1 ± 2.8 mV, at pH 2.6) due to interfacial amines (Figure S54), cationic GPC columns were selected to minimize CND/column interactions. Figure 2a shows representative GPC elution profiles, with signals recorded by RI (red), UV absorption at 330 nm (purple), right‐angle light scattering (green), and viscometer differential pressure (viscometer–DP, blue). The UV trace revealed a single unimodal peak, indicating absence of molecular impurities. This result was corroborated by the alignment of peaks across all detectors, confirming that the primary eluting species corresponded exclusively to CNDs. High signal reproducibility was observed across replicate injections (Figure S57).

Quantitative analysis of the elution profile yielded a number‐average molecular weight (*M_n_*) of 3927 ± 8 g mol^−1^, a weight‐average molecular weight (*M_w_*) of 5272 ± 12 g mol^−1^, and a dispersity (*Đ*) of 1.34, indicating a rather monodisperse CND population. This was confirmed by the hydrodynamic diameter determined using the viscometer detector 2.8 ± 0.2 nm, which was consistent with the DLS data 3.8 ± 0.2 nm, and AFM diameter 2.6 ± 0.1 nm. Importantly, sample recovery based on full integration of the UV trace acquired at 330 nm was nearly quantitative (99 wt%), confirming minimal presence of residual small molecules.

Interestingly, despite our rigorous purification protocol, GPC analyses across multiple synthesis batches revealed some degree of variation in the RI signal of CNDs at 18.9 mL of retention volume and of molecular impurity at 21.5 mL. While most batches exhibited a clean, unimodal profile for the CND peak at 18.9 mL (e.g., batch A, Figure 2b, red curve), others (batches B and C, blue and green curves in Figure 2b, respectively) displayed a secondary shoulder or peak. This suggests the presence of slightly smaller CND populations, often accompanied by an increased impurity peak at 21.5 mL (Figure 2b, blue and green curves). MD‐GPC analysis of these batches revealed *M_n_* values of 4815 ± 54 g mol^−1^ (batch B) and 4547 ± 34 g mol^−1^ (batch C), and *M_w_* values of 7035 ± 85 g mol^−1^ and 6710 ± 45 g mol^−1^, respectively, with recoveries still above 95 wt% (Table S1). Across all batches, the averaged values were *M_n_* = 4400 ± 458 g mol^−1^ and *M_w_* = 6333 ± 907 g mol^−1^, with impurity content remaining below 5 wt%, even after extensive dialysis, suggesting minor yet reproducible batch‐to‐batch variation.

^1^H NMR spectra (Figure 2c) of batches B and C further confirmed the presence of small‐molecule contaminants, evident from the sharp multiplets in the 4.5–2.5 ppm range, which were absent in batch A. DOSY NMR (Figure S58) also identified these signals as fast‐diffusing species, distinct from the CND population.

Determination of molecular weight by MD‐GPC also enabled the determination of the number surface functionalities per CND. Given the importance of primary amines in CND conjugation chemistry and catalysis, we used the Kaiser test to quantify these groups. By combining Kaiser test results (in µmol –NH_2_ g^−1^ material) with the *M_n_*, we calculated an average of 7 ± 1 primary aliphatic amines per CND under standard conditions (120 °C; Figure S59).

To further assess interfacial reactivity, CNDs were allowed to react with 4‐fluorobenzaldehyde to form fluorinated imines, which were monitored by ^19^F NMR spectroscopy using trifluorotoluene as an internal standard (Figure S60).^[^ ^29^ ^]^ The appearance of multiplets at ∼110 ppm confirmed imine formation, consistent with a fluorinated model compound and previous literature reports.^[^ ^30^ ^]^ A 24 h kinetic study revealed quantitative reaction of all amines on the CNDs under the applied conditions (Figure S60).

Together, these results establish a predictive and quantitative framework for surface functionalization of CNDs. The ability to precisely determine molecular weight, dispersity, and surface amine content positions these materials as promising, tunable platforms for advanced nanostructure construction. Moreover, these data offer new intriguing opportunities in the rational design and development of new nano‐aminocatalysts based on CNDs.

## Conclusions

In this work, we present a comprehensive investigation into the early stages of CND formation, combining synthetic, analytical, and spectroscopic methodologies to gain molecular‐level insight into their formation mechanism. By isolating and characterizing key intermediates generated from the hydrothermal treatment of arginine and ethylenediamine, we unveil the chemical transformations that occur prior to particle formation. These species, which had not previously been structurally confirmed within the CND synthetic landscape, were identified through a combination of chromatographic separation, NMR spectroscopy, and mass spectrometry. Their structures were further validated *via* independent synthesis and comparison with authentic standards.

Fully developed CNDs were subsequently isolated and subjected to extensive physicochemical characterization, confirming high sample purity, narrow dispersity, and consistent structural features across batches. The use of multi‐detection gel permeation chromatography not only enabled the absolute determination of molecular weight and size distribution providing critical insight into the macromolecular nature of CNDs, but also allowed for the quantification of molecular impurities, which in highly pure samples should represent <5% of the total composition. Complementary measurements using AFM, DLS, and NMR spectroscopy supported these findings and reinforced the reliability of our purification and characterization strategy.

A particularly significant advancement is the quantification of surface amine groups per CND (equal to 7 ± 1), which was accomplished from the *M_n_* using both the Kaiser test and ^19^F NMR after derivatization with a fluorinated aldehyde. This level of surface characterization, when combined with precise size and molecular weight data, paves the way for rational conjugation strategies and functional applications of CNDs in nanotechnology, catalysis, and biomedicine.

Altogether, this work bridges the gap between organic reaction mechanisms and nanomaterial design, offering a rigorous and reproducible workflow for the mechanistic understanding and functional evaluation of carbon nanodots. It sets the stage for high precision and purpose‐driven synthesis of CNDs, ultimately contributing to their development as highly tunable nanoplatforms.

## Conflict of Interests

The authors declare no conflict of interest.

## Supporting information



Supporting Information

## Data Availability

The data that support the findings of this study are available in the supplementary material of this article.
