# Supplementary material for: Molecular Insights into the Formation and Functionalization of Carbon Nanodots: From Precursor Intermediates to Surface Chemistry Quantification
Source: Angew Chem Int Ed Engl. 2025 Aug 8;64(39):e202515073. doi: 10.1002/anie.202515073 (PMC12455451; doi:10.1002/anie.202515073)
Supplement: Supplementary file 1 — Supporting Information [file ANIE-64-e202515073-s001.docx]

**Supporting Information**

**Molecular Insight into the Formation and Functionalization of Carbon Nanodots: From Precursor Intermediates to Surface Chemistry Quantification**

Emanuele Giuliani,^+^ Maria Sbacchi,^+^ Serena Agostini, Giacomo Filippini, Beatrice Bartolomei,* Pierangelo Gobbo,* Maurizio Prato*

**Table of Contents**

1. **General information 2**
2. **Compounds isolated from CND synthesis 4**
   1. CNDs (Arg/EDA), fraction #1 5.9 min

2.2 CNDs (Arg/EDA), fraction #2 9.3 min

2.3 CNDs (Arg/EDA), fraction #3 10.3 min

2.4 CNDs (Arg/EDA), fraction #4 10.6 min

1. **Direct synthesis of target compounds 25**

3.1 Compound **1a**

3.2 Compound **1b**

3.3 Compound **2**

3.4 Compound **3**

3.5 Compound **4**

1. **Assessment of CND purity and determination of the molecular weight 56**
   1. Characterization of CNDs
   2. Determination of CND molecular weight by MD-GPC

4.3 Quantification of CND primary aliphatic amines by Kaiser Test

4.4 Quantification of CND primary amines by ^19^F NMR spectroscopy

1. **References 62**
2. **General information**

**Synthesis.** Chemicals were purchased from Sigma Aldrich and were used as received unless otherwise stated. Solvents were purchased from Sigma Aldrich, while deuterated solvents were purchased from VWR. Ultrapure fresh H_2_O obtained from a Millipore H_2_O purification system (>18MΩ Milli-Q, Millipore) was used in all experiments. Microwave synthesis was performed on a CEM Discover-SP, using 10 mL glass microwave vials. Merck Omnipore 0.1 μm PTFE Membrane filters (25 mm) were employed for filtration. Solutions were concentrated under reduced pressure on a Büchi rotatory evaporator or freeze-dried using CoolSafe 9L; Labogene, model no. CoolSafe-9 lyophilizer. **Purification.** Semipreparative HPLC separation was carried out employing Agilent 1260 Infinity. A Phenomenex C8 column (250x10 mm) with 5 um pore size and Kinetex C18 column (250 x 10 mm) with 5 µm pore size were used for HPLC separation. The UV-Vis chromatograms were monitored at 215 nm and 254 nm. The mobile phase was prepared using binary mixtures of acetonitrile (ACN) and H_2_O (containing 1% of HCOOH). The flow rate of the mobile phase was set at 2 mL/min. The eluted gradient was programmed as the percentage of acetonitrile was linearly increased, after 4 min, from 0 to 100% in 41 min. The injection volume was 50 µL, and the column temperature was maintained at 40 °C. Preparative HPLC separation was carried out employing Shimadzu CBM-20A. A Preparative Gemini NX-C18 column (250x21.2 mm) with 5 µm pore size was used for HPLC separation. The UV-Vis chromatograms were monitored at 215 nm. The mobile phase was prepared using binary mixtures of ACN and H_2_O (containing 0.05% of trifluoroacetic acid). The flow rate of the mobile phase was set at 10 mL/min. The eluted gradient was programmed as the percentage of acetonitrile was linearly increased, after 4 min, from 0 to 100% in 41 min. The injection volume was 1 mL. **Characterization.** ^1^H NMR spectra and DOSY experiments were recorded on a Varian Inova spectrometer (^1^H = 500 MHz) equipped with Performa II-Z gradient coils with Absolute Value – gradient compensated stimulated Echo gHMBC. Chemical shifts were reported in ppm using the solvent residual signal as an internal reference (D_2_O: *δH* = 4.79 ppm). ^19^F NMR spectra were recorded on Varian 400 MHz spectrometer (^19^F = 376 MHz). All spectra were recorded at 25 °C. All spectra were recorded at 25 °C and analyzed with MestReNova v12.0.1-20560. High-resolution mass spectra (HRMS) were obtained on Bruker micrOTOF-Q (ESI-TOF). AFM images were obtained with a Nanoscope IIIa (VEECO Instruments) using tapping mode with a HQ:NSC19/ALBS probe (80 kHz; 0.6 N m^-1^) (MikroMasch). Samples were prepared by drop-casting methanol solutions (concentration around 1 ng mL^-1^) on an exfoliated mica substrate. The obtained micrographs were analyzed with Gwyddion 2.63. Zeta potential and DLS measurements were performed on a Zetasizer Ultra Red (Malvern Panalytical Ltd) using a ζ-Potential cuvette (Malvern Panalytical Ltd, model DTS1070) and a low-volume quartz batch cuvette (Malvern Panalytical Ltd, model ZEN2112), respectively. CND samples were prepared at a concentration of *ca.* 5 mg mL^-1^ in a pre-filtered sodium nitrate solution (0.1 M NaNO_3_ + 0.5 v/v% acetic acid pH 2.6). Before measurement, sample solutions were also filtered through a 0.45 µm regenerated cellulose syringe filter. Attenuated Total Reflectance (ATR) Fourier-Transform Infrared spectroscopy was performed on a Shimadzu IRAffinity1S equipped with a QATR-10 with diamond crystal. Samples were analyzed as powders by placing them on the crystal and pressing them to record the spectrum. Absorption spectra were collected with an Agilent Cary 5000 UV-Vis spectrophotometer. Spectra were acquired at a concentration of 1 mg mL^-1^ in MilliQ water at room temperature, using 10 mm path-length quartz cuvettes. Fluorescence spectra were recorded on an Edinburgh instruments FS5 spectrofluorometer using a 150 W CW Ozone-free xenon arc lamp as the source and a Photomultiplier R928P (spectral coverage: 200 nm – 900 nm) as detector. All spectra were registered with an absorbance equal to 0.1 in MilliQ water at room temperature using 10 mm path-length quartz cuvettes. Multi-detection gel permeation chromatography (MD-GPC) measurements were performed on an OMNISEC RESOLVE/OMNISEC REVEAL system from Malvern Panalytical Ltd. The OMNISEC RESOLVE system comprises an isocratic pump with continuous back seal washing that operates at 0.5 mL min^-1^ of flowrate a degasser with > 90% degassing capacity, a thermostated autosampler (4 °C) with a 250 µL glass syringe and a 300 µL injection loop, a thermostated column oven (20 °C) containing two Tosoh Bioscience TSKgel GMPWXL-CP (G6000 + G3000PWXL-CP) cationic size exclusion chromatography (SEC) columns connected in series. The OMNISEC REVEAL comprises four thermostated detectors (20 °C): (1) Refractive index deflection detector operating at 640 nm and with a 12 µL reference cell; (2) Diode-array-based UV-Vis spectrophotometer working between 190 – 900 nm (accuracy < 1 nm) and equipped with 7.5 µL cell with a 10 mm path length; (3) Light scattering detector comprising a Right Angle Light Scattering detector positioned at 90° to the sample flow, and a Low Angle Light Scattering detector positioned at 7° to the sample flow, the light source is a 640 nm laser (50 mW), the cell volume is 18 µL; (4) 4-capillary Wheatstone bridge viscometer with self-balancing mechanism, user-exchangeable capillaries and one delay column. A typical run was performed using 0.1 M NaNO_3_ + 0.5 v/v% acetic acid – to reach pH 2.6 – as mobile phase, which was pre-filtered through a bottle vacuum filter funnel with a 0.22 µm PES membrane (Steritop^®^, Merck). CND samples were prepared at a concentration of *ca.* 5 mg mL^-1^, dissolved in the eluent, and filtered through 0.45 µm regenerated cellulose syringe filters.

1. **Compounds isolated from CND synthesis**

A solution of *L*-arginine (87.0 mg, 0.5 mmol), ethylenediamine (33.0 µL, 0.5 mmol), and Milli-Q H_2_O (100.0 μL) was heated at 250 °C, 26 bar and 200 W from 1 to 12 cycles of 15 s heating and 5 s cooling. The reaction crudes were filtered on a 0.2 μm PTFE filter and diluted in a vial with 2 mL of Milli-Q H_2_O. Then, the mixtures were analyzed through ^1^H NMR and Semipreparative C8-HPLC. The same procedure was followed using *L*-arginine (87.0 mg, 0.5 mmol) and Milli-Q H_2_O (100.0 μL).

**Figure S1.** Semipreparative C8-HPLC chromatograms monitored at 215 nm of the reaction crudes between 1-12 cycles of heating from L-arginine and ethylenediamine.

**Figure S2.** Semipreparative C8-HPLC chromatograms monitored at 215 nm of the reaction crudes between 1-12 cycles of heating from *L*-arginine.

- 1. **CNDs (Arg/EDA), fraction #1 5.9 min**

**
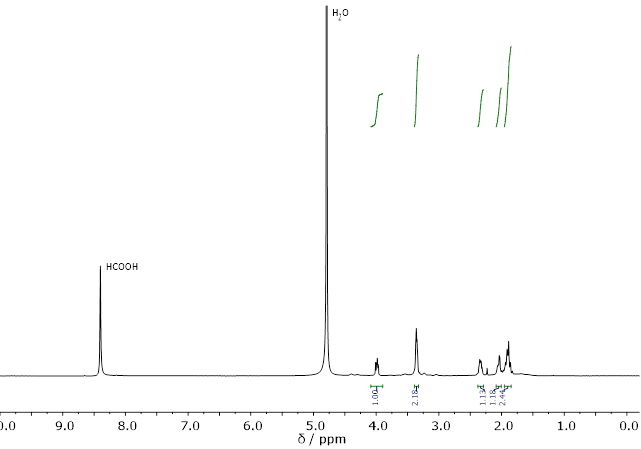
**

**Figure S3.** Fraction **1**: ^1^H NMR (D_2_O, 400 MHz, r.T.)

**
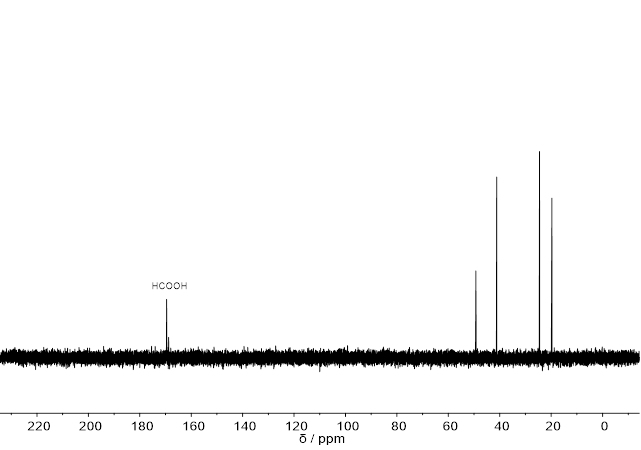
**

**Figure S4.** Fraction **1**: ^13^C NMR (D_2_O, 101 MHz, r.T.)

**
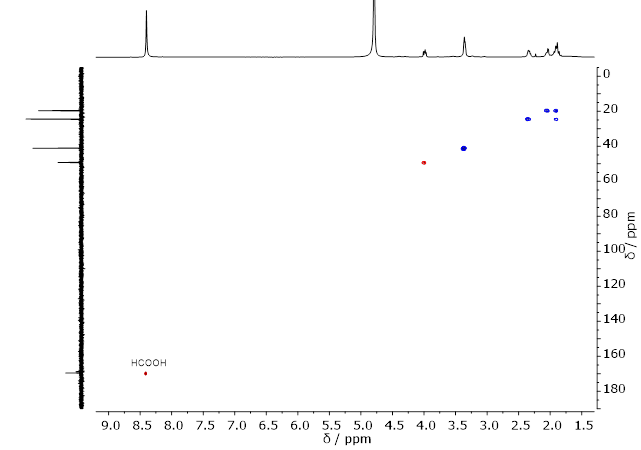
**

**Figure S5.** Fraction **1**: HSQC NMR (D_2_O, 400 MHz, r.T.)

**Figure S6.** Fraction **1**: HRMS, experimental spectra (top), simulated spectra (bottom).

- 1. **CNDs (Arg/EDA), fraction #2 9.3 min**

**
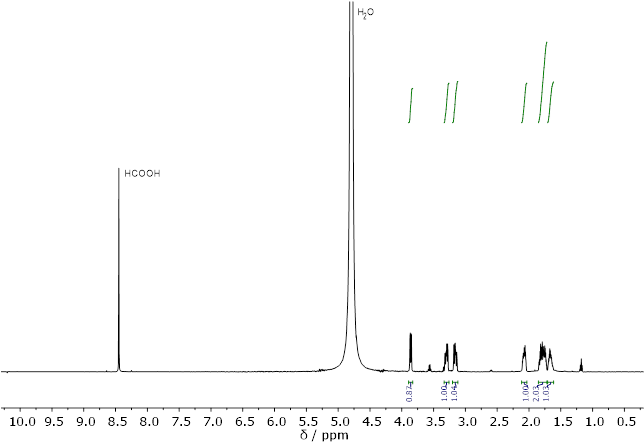
**

**Figure S7.** Fraction **2**: ^1^H NMR (D_2_O, 400 MHz, r.T.)

**
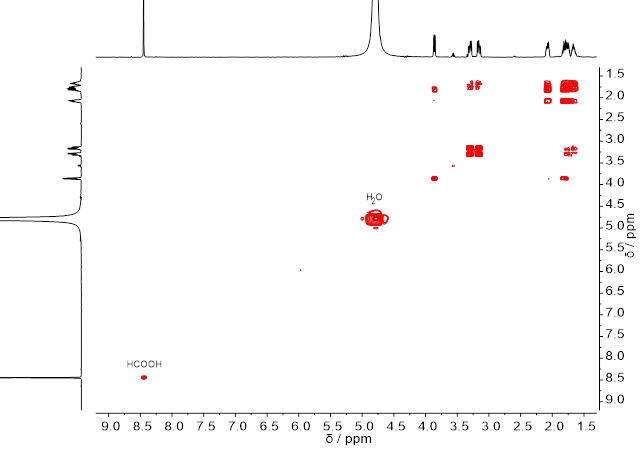
**

**Figure S8.** Fraction **2**: COSY NMR (D_2_O, 400 MHz, r.T.)

**
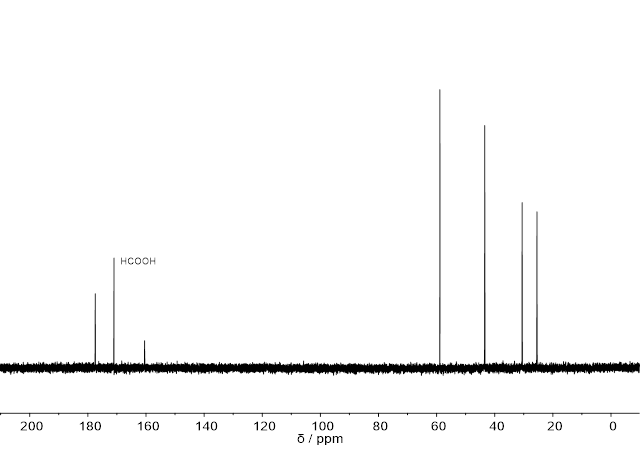
**

**Figure S9.** Fraction **2**: ^13^C NMR (D_2_O, 101 MHz, r.T.)

**
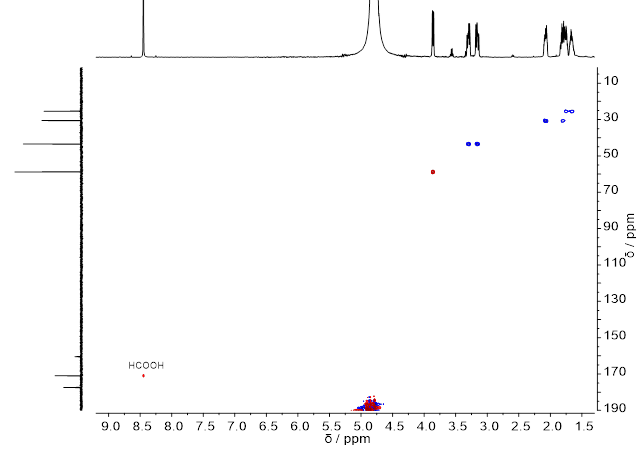
**

**Figure S10.** Fraction **2**: HSQC NMR (D_2_O, 400 MHz, r.T.)

**Figure S11.** Fraction **2**: HRMS, experimental spectra (top), simulated spectra (bottom).

- 1. **CNDs (Arg/EDA), fraction #3 10.3 min**

**
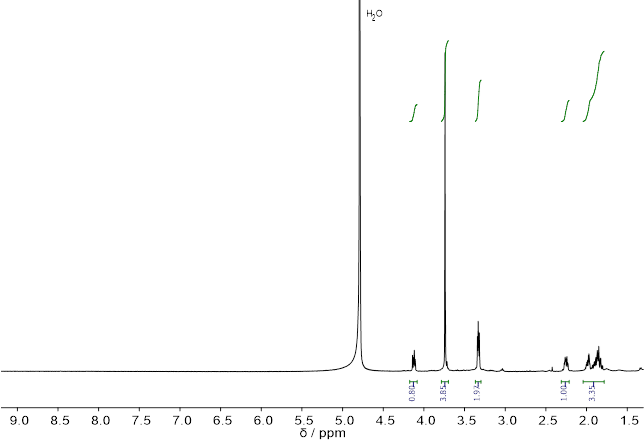
**

**Figure S12.** Fraction **3**: ^1^H NMR (D_2_O, 500 MHz, r.T.)

**
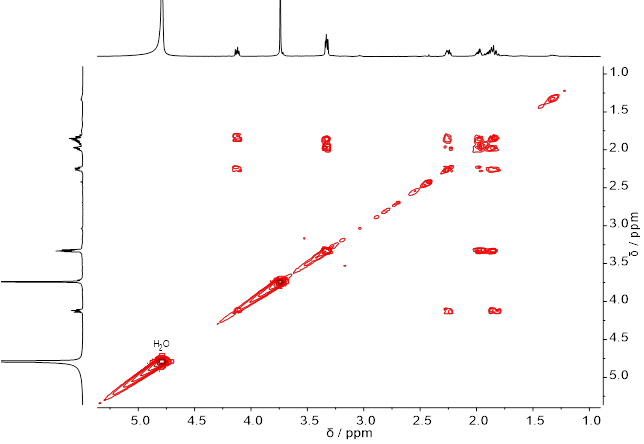
**

**Figure S13.** Fraction **3**: COSY NMR (D_2_O, 500 MHz, r.T.)

**
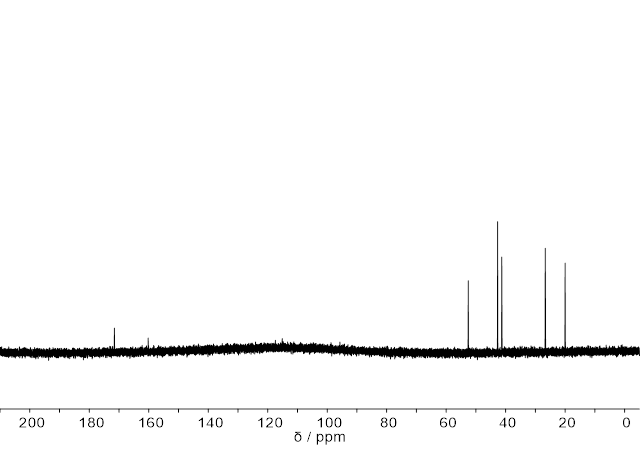
**

**Figure S14.** Fraction **3**: ^13^C NMR (D_2_O, 126 MHz, r.T)

**
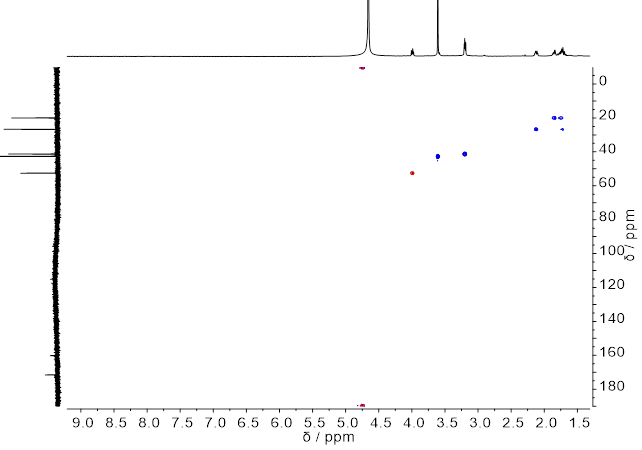
**

**Figure S15.** Fraction **3**: HSQC NMR (D_2_O, 500 MHz, r.T.)


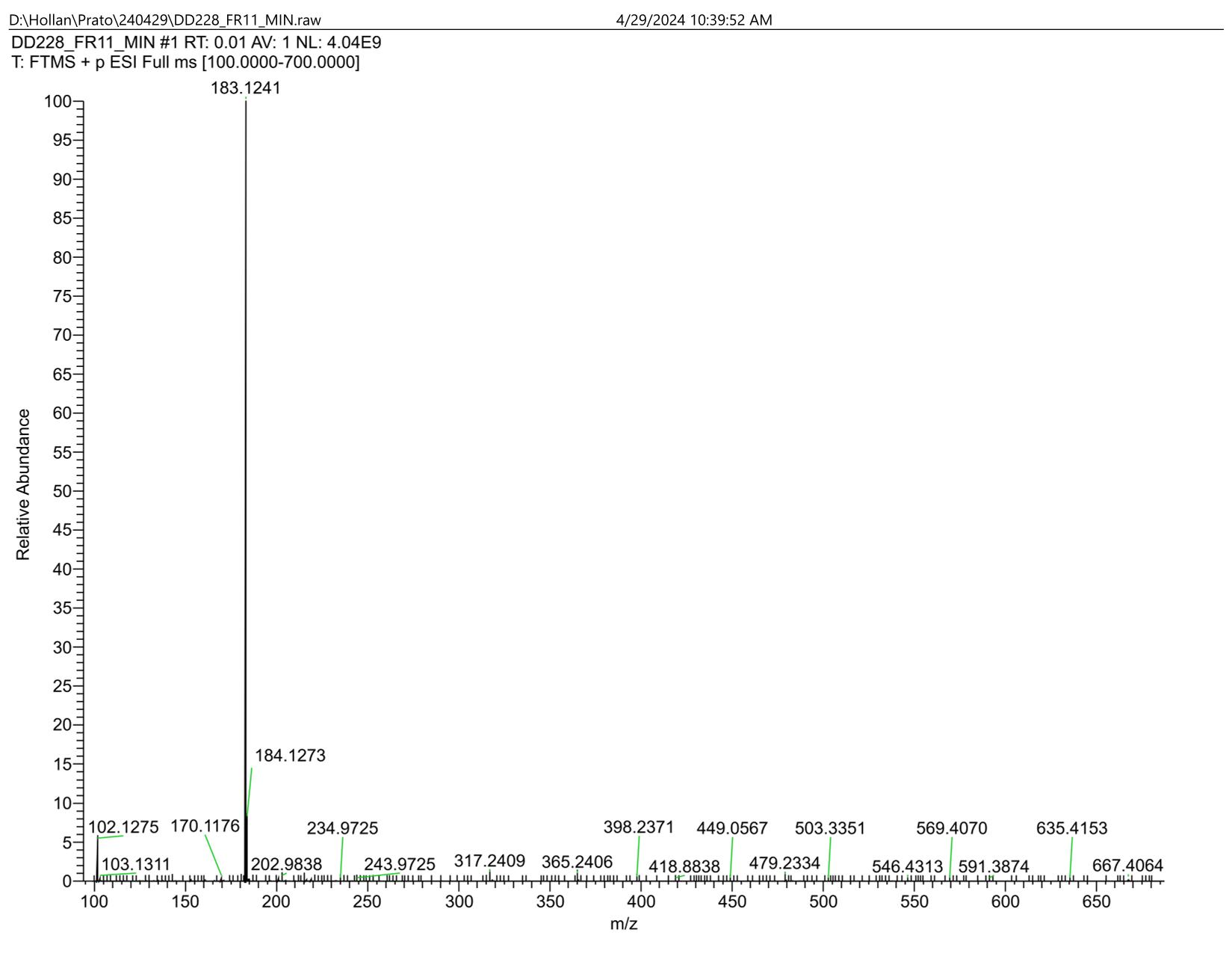


**Figure S16.** Fraction **3**: HRMS spectrum.

- 1. **CNDs (Arg/EDA), fraction #4 10.6 min**

Fraction **4** could not be entirely separated under the employed semipreparative HPLC conditions. An additional Semipreparative C18-HPLC separation was performed. The combined fractions were freeze-dried.***
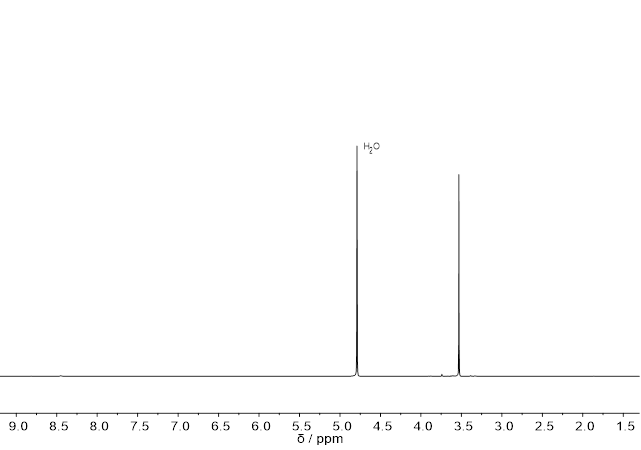
***

**Figure S17.** Fraction **4**: ^1^H NMR (D_2_O, 500 MHz, r.T.)

**
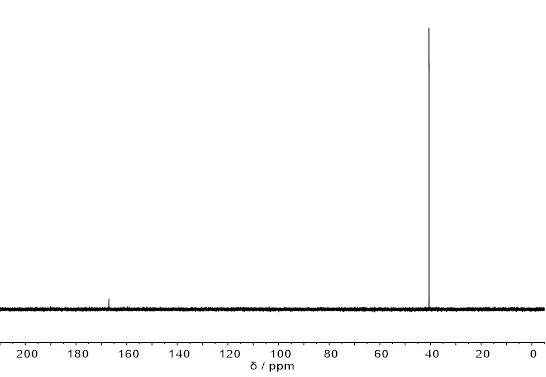
**

**Figure S18.** Fraction **4**: ^13^C NMR (D_2_O, 101 MHz, r.T.)

**
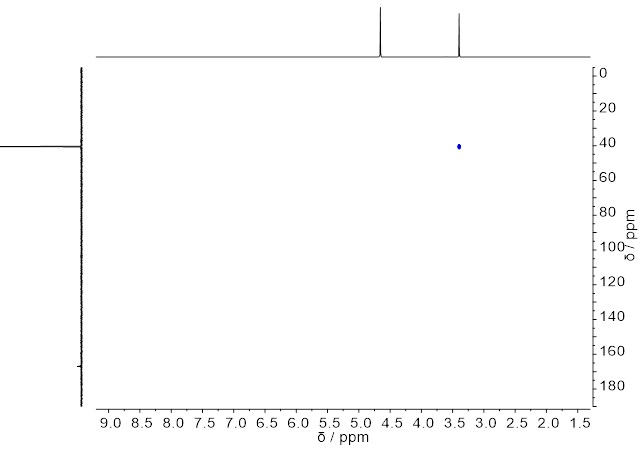
**

**Figure S19.** Fraction **4**: HSQC NMR (D_2_O, 500 MHz, r.T.)

**
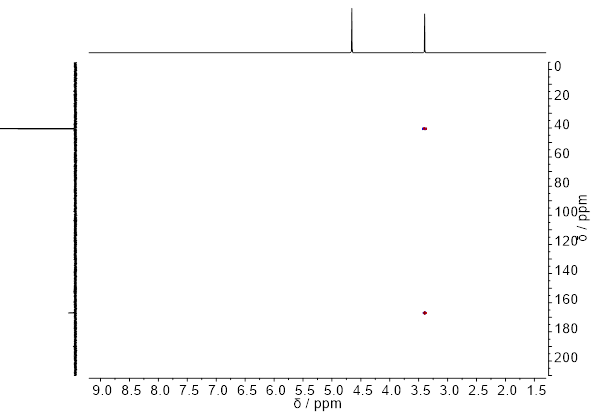
**

**Figure S20.** Fraction **4**: HMBC NMR (D_2_O, 500 MHz, r.T.)


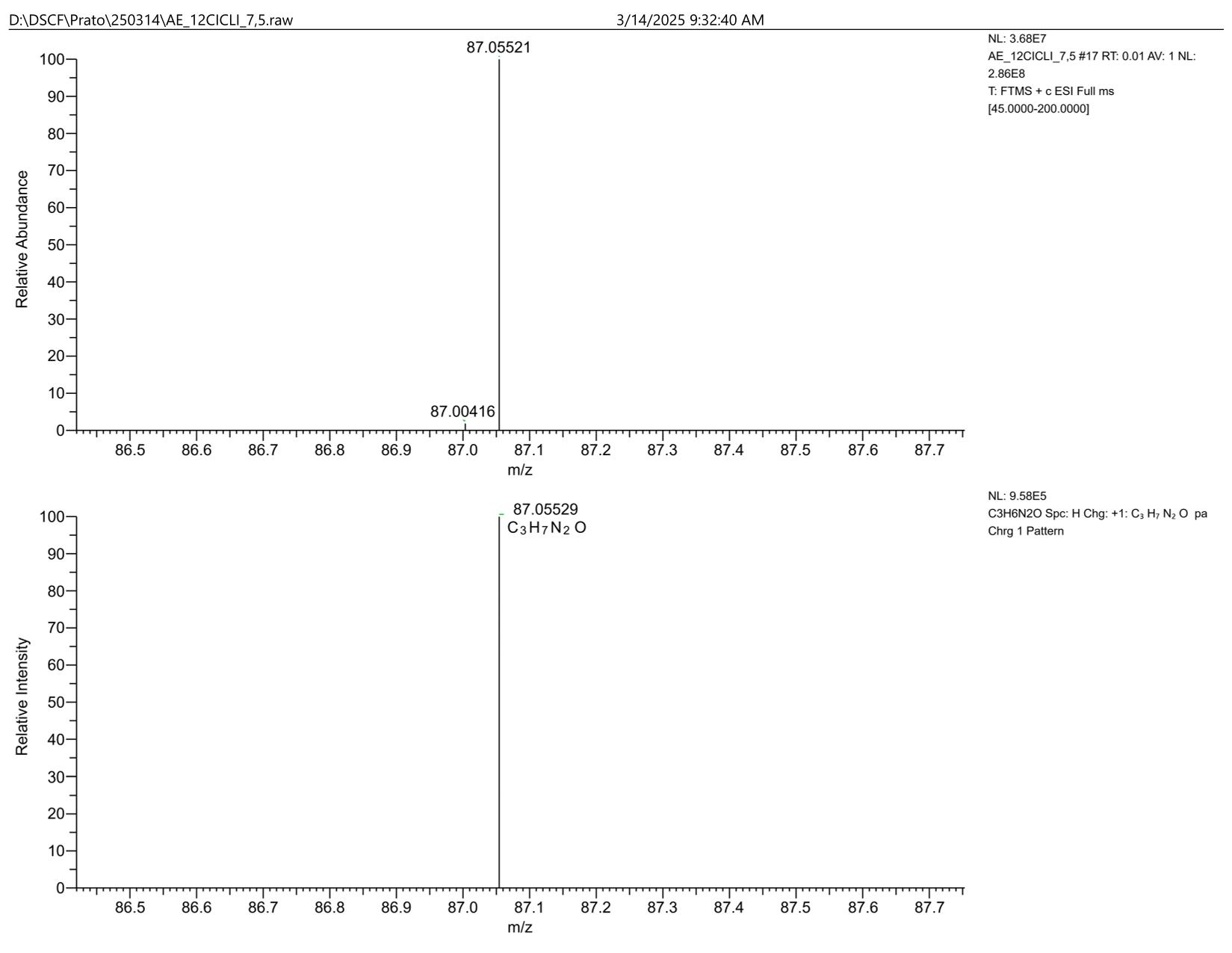
**Figure S21.** Fraction **4**: HRMS, experimental spectra (top), simulated spectra (bottom).

1. **Direct synthesis of target compounds**
   1. **Compound 1a:** carbamimidoylproline


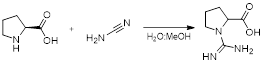


Prepared according to a modified literature procedure.^[1]^ *L*-proline (3.0 g, 26 mmol) was dissolved in 11 mL of H_2_O, with the pH of the solution adjusted to 8.5 using NaOH (1 M). Following, MeOH (6 mL) was added to the solution, and a 20% aqueous solution of cyanamide (1.3 g, 31 mmol) was then introduced dropwise over 2 hrs while refluxing. The mixture was refluxed for 2 hrs and then stirred at room temperature overnight. The solvent was then evaporated, and the residue was precipitated in isopropyl alcohol and recrystallized from H_2_O (2.8 g, 15.6 mmol, 60% yield).

**^1^H NMR** (500 MHz, D_2_O) δ 4.20 (dd, J = 8.7, 3.1 Hz, 1H), 3.51 (ddd, J = 9.5, 8.0, 3.9 Hz, 1H), 3.42 (td, J = 9.0, 7.3 Hz, 1H), 2.34 – 2.21 (m, 1H), 2.15 – 2.05 (m, 1H), 2.05 – 1.86 (m, 2H). **^13^C NMR** (126 MHz, D_2_O) δ 178.64, 156.16, 62.25, 47.61, 31.03, 23.38. **HRMS** (ESI, positive mode): *m/z* calcd. for [C_8_H_17_N_4_O_2_]^+^: 201.13460, found: 201.13441.

**
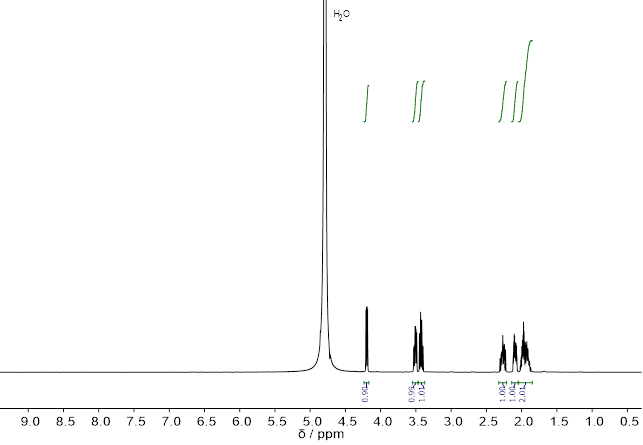
**

**Figure S22.** Compound **1a**: ^1^H NMR (D_2_O, 500 MHz, r.T)

**
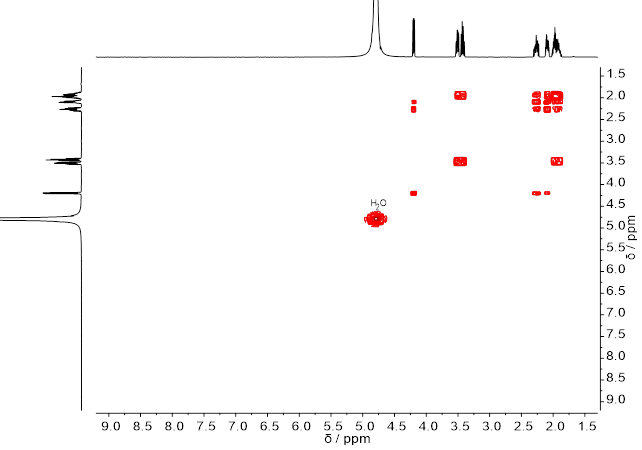
**

**Figure S23.** Compound **1a**: COSY NMR (D_2_O, 500 MHz, r.T.)


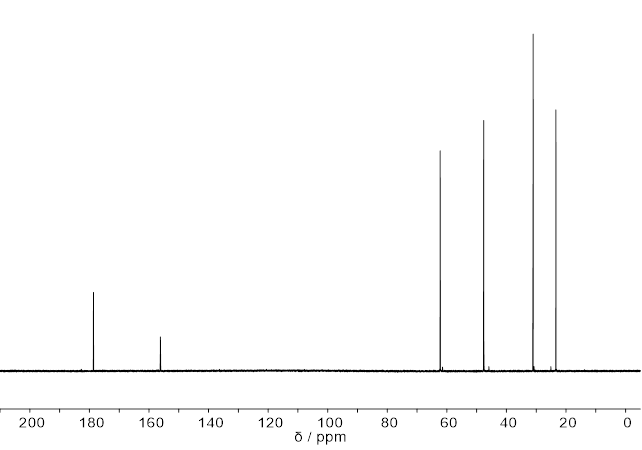


**Figure S24.** Compound **1a**: ^13^C NMR (D_2_O, 126 MHz, r.T.)


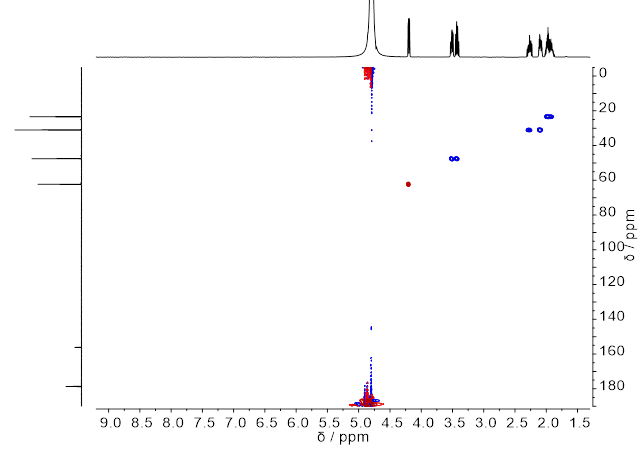


**Figure S25.** Compound **1a**: HSQC NMR (D_2_O, 500 MHz, r.T.)


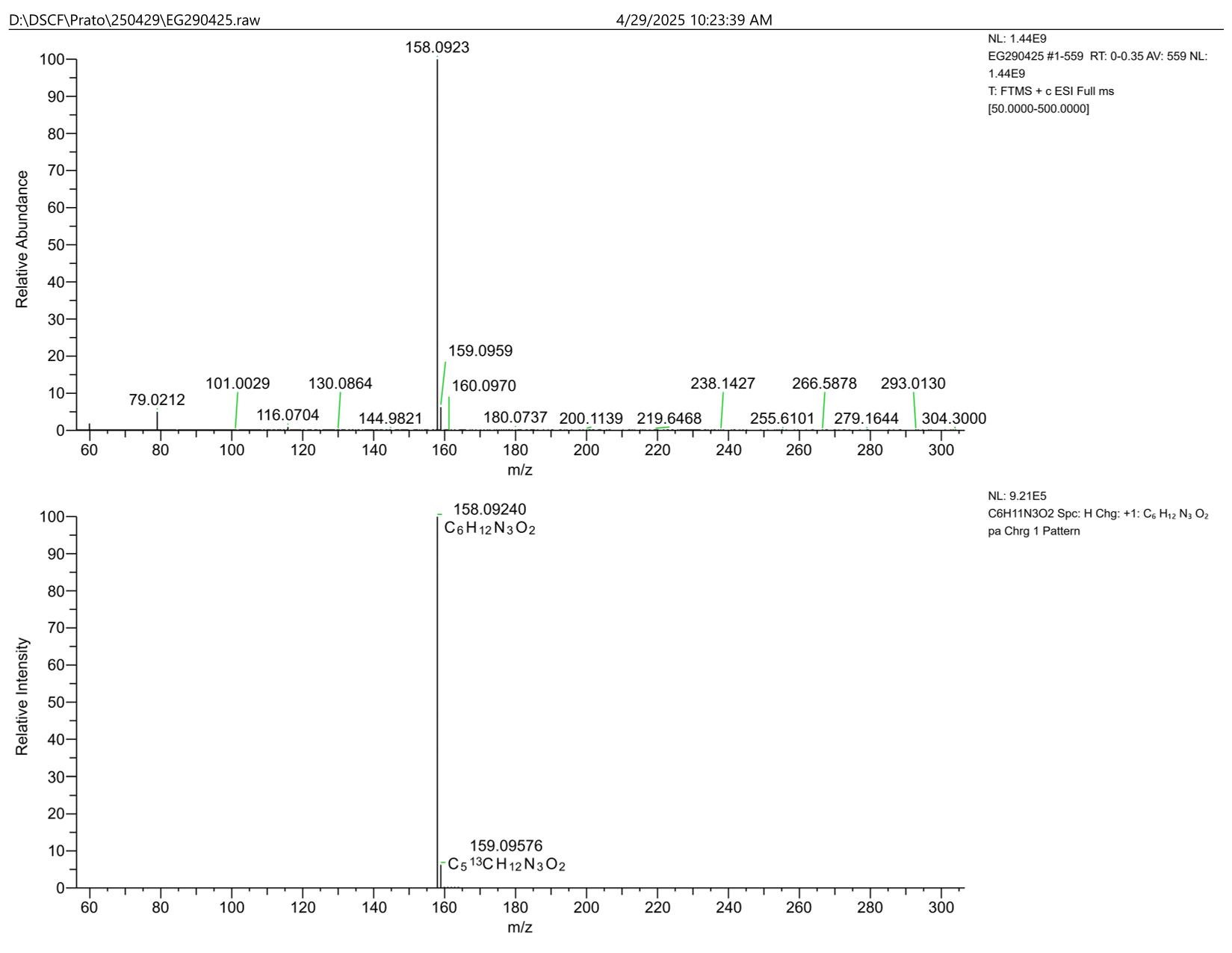


**Figure S26.** Compound **1a**: HRMS, experimental spectra (top), simulated spectra (bottom).


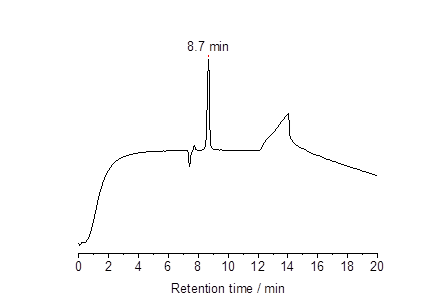


**Figure S27.** Compound **1a**: Semipreparative C8-HPLC chromatogram monitored at 215 nm.

- 1. **Compound 1b:** 2-imino-1,3-diazepane-4-carboxylic acid


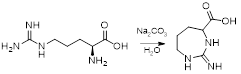


Prepared according to a modified literature procedure.^[2]^ *L*-arginine (5 g, 28 mmol, 1 eq.) was dissolved in H_2_O (30 mL), then Na_2_CO_3_ (7.4 g, 70 mmol, 2.5 eq.) was added at room temperature, and the resulting mixture was stirred at 90°C for 12 hrs. The solution was cooled down to room temperature and acidified to pH 1 by adding HCl (>37%) to induce the precipitation of the product. The precipitate was filtered and dried to give **1b** (300 mg, 1.7 mmol, 6% yield).

**^1^H NMR** (400 MHz, D_2_O) δ 3.84 (dd, *J* = 9.1, 2.8 Hz, 1H), 3.33 – 3.24 (m, 1H), 3.19 – 3.10 (m, 1H), 2.13 – 2.00 (m, 1H), 1.86 – 1.58 (m, 3H); **^13^C NMR** (101 MHz, D_2_O) δ 177.66, 160.87, 58.88, 43.48, 30.73, 25.63. **HRMS** (ESI, positive mode): *m/z* calcd. for [C_6_H_11_O_2_N_3_]^+^: 158.0924; found: 158.0924.

**
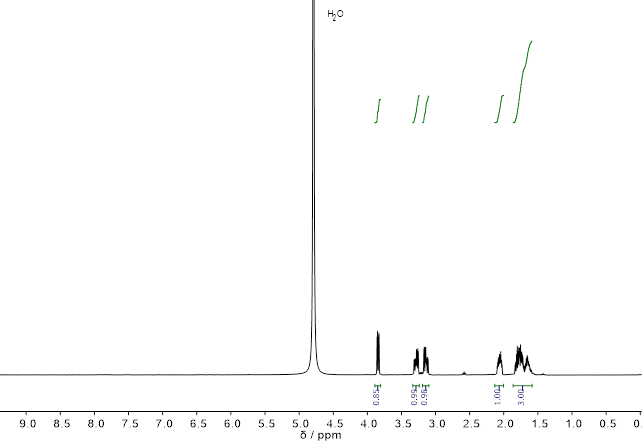
**

**Figure S28.** Compound **1b**: ^1^H NMR (D_2_O, 400 MHz, r.T.)

**
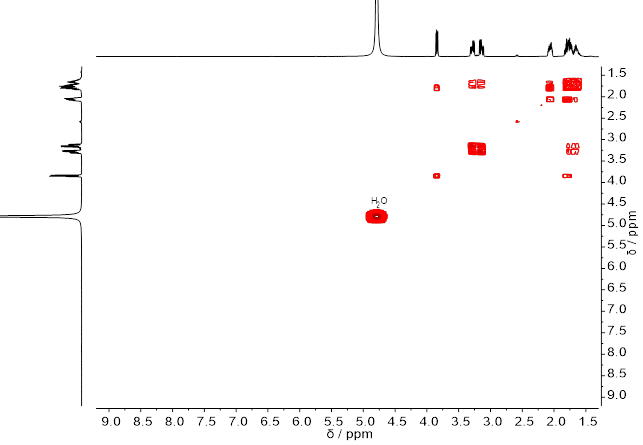
**

**Figure S29.** Compound **1b**: COSY NMR (D_2_O, 400 MHz, r.T.)

*
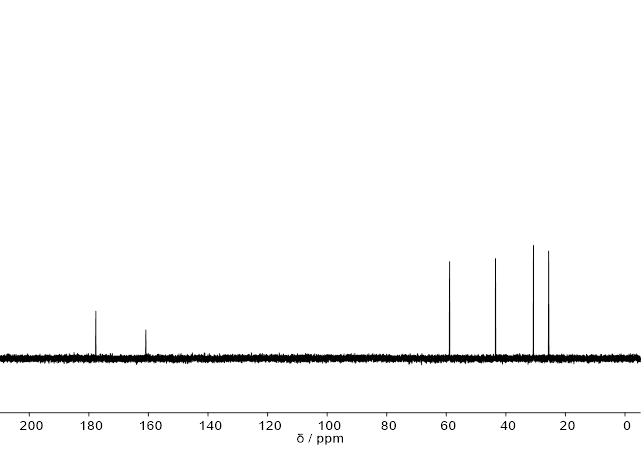
*

**Figure S30.** Compund **1b**: ^13^C NMR (D_2_O, 101 MHz, r.T.)

**
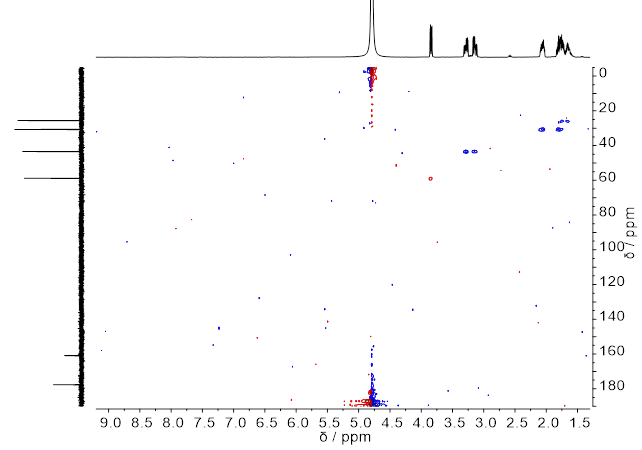
**

**Figure S31.** Compound **1b**: HSQC NMR (D_2_O, 400 MHz, r.T.)

**Figure S32.** Compound **1b**. HRMS, experimental spectra (top), simulated spectra (bottom)


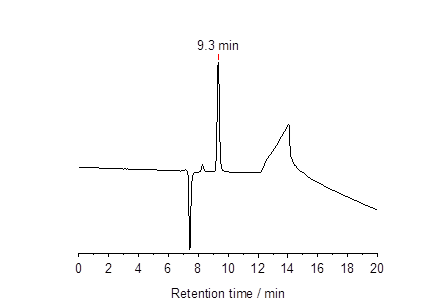


**Figure S33.** Compound **1b**: Semipreparative C8-HPLC chromatogram monitored at 215 nm.

- 1. **Compound 2:** 3-aminopiperidin-2-one

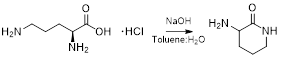


Prepared according to a modified literature procedure.^[3]^ *L*-ornithine monohydrochloride (2 g, 12 mmol) was added to a stirred solution of NaOH (500 mg, 12 mmol) in H_2_O (2 mL) at room temperature. After 15 min, this solution was added to a stirred mixture of alumina (6 g) and toluene (20 mL) and heated under reflux for 3 hrs. The H_2_O produced during the reaction was collected in a Dean-Stark trap. The reaction mixture was allowed to cool down at room temperature and then alumina was filtered off and washed with 10% MeOH/CH_2_Cl_2_. The filtrate was recovered and the solvent was removed under reduced pressure to give **2** (1.2 g, 10.5 mmol, 88% yield).

**^1^H NMR** (500 MHz, D_2_O/ HCOOH) δ 3.96 (dd, *J* = 11.3, 6.2 Hz, 1H), 3.33 (q, *J* = 4.4 Hz, 2H), 2.39 – 2.24 (m, 1H), 2.09 – 1.96 (m, 1H), 1.96 – 1.79 (m, 2H). **^13^C NMR** (126 MHz, D_2_O/ HCOOH) δ 168.74, 49.26, 41.16, 24.50, 19.70. **HRMS** (ESI, positive mode) m/z calcd. for C_5_H_10_N_2_O [M+Na]^+^: 137.0685, found: 137.0687.

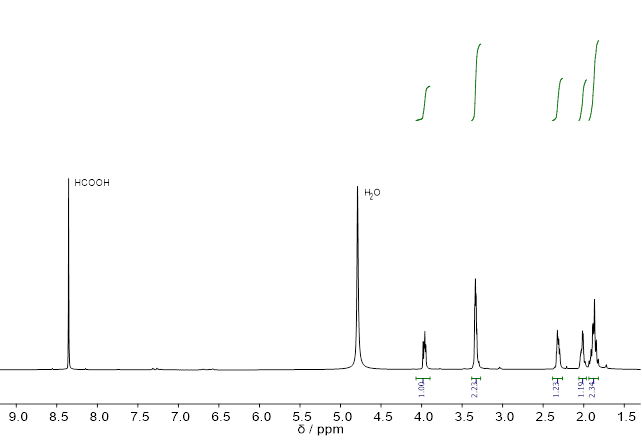


**Figure S34.** Compound **2**: ^1^H NMR (D_2_O/HCOOH, 500 MHz, r.T.)

**
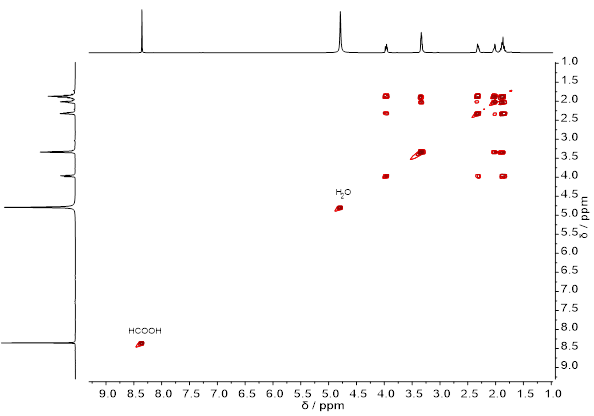
**

**Figure S35.** Compound **2**: COSY NMR (D_2_O/HCOOH, 500 MHz, r.T.)

**
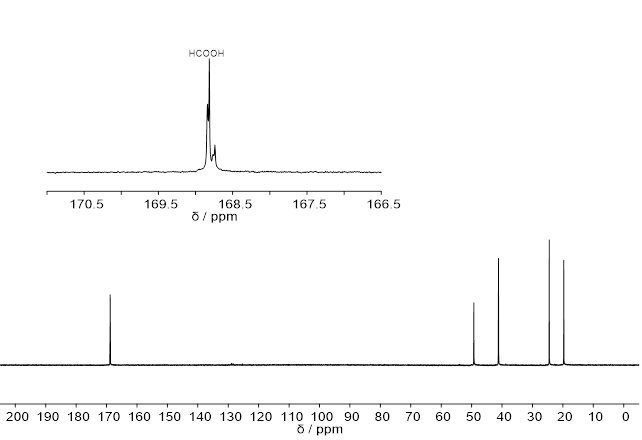
**

**Figure S36.** Compound **2**: ^13^C NMR (D_2_O/HCOOH, 126 MHz, r.T.)


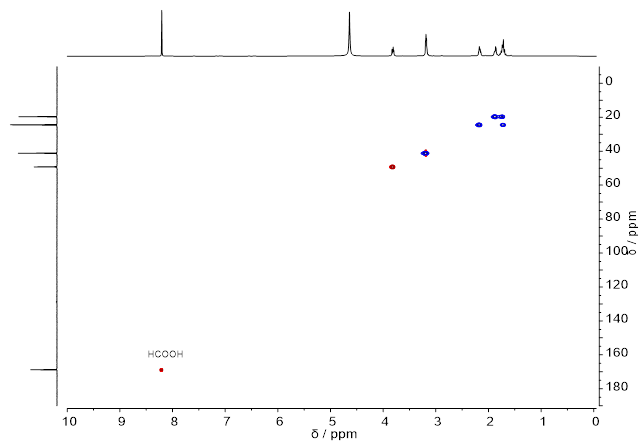


**Figure S37.** Compound **2**: HSQC spectra (D_2_O/HCOOH, 500 MHz, r.T.)

**Figure S38.** Compound **2**: HRMS, experimental spectra (top), simulated spectra (bottom).


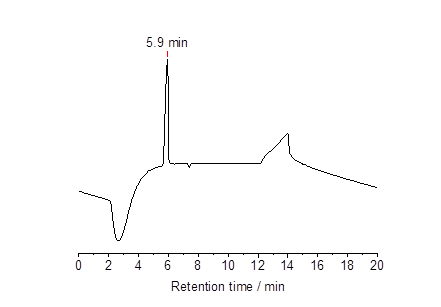


**Figure S39.** Compound **2**: Semipreparative C8-HPLC chromatogram monitored at 215 nm.

- 1. **Compound 3:** 2-imidazolidinone

**
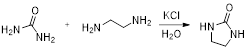
**

Prepared according to a modified literature procedure.^[4]^ Ethylenediamine (33 µL, 0.5 mmol, 1 eq.), urea (50 mg, 0.83 mmol, 1,7 eq.), and KCl (20 mg) were dissolved in 100 μL of H_2_O and heated in MW for 3 min at 240°C. The obtained solution was diluted in 1 mL of Milli-Q H_2_O and purified by semipreparative C8-HPLC. Compound **3** was obtained as a white solid after freeze-drying of the collected fraction (7 mg, 0.8 mmol, 16% yield).

**^1^H NMR** (500 MHz, D_2_O) δ 3.53 (s, 1H). **^13^C NMR** (126 MHz, D_2_O) δ 167.03, 40.60**. HRMS** (ESI, positive mode): m/z calcd. for [C_3_H_7_N_2_O]^+^: 87.05529, found: 87.0553.


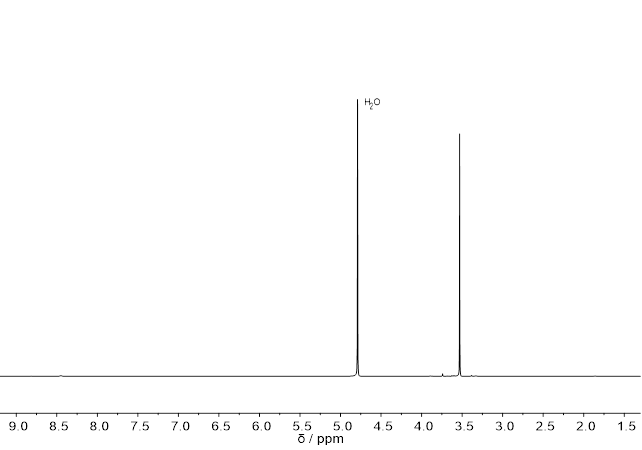


**Figure S40.** Compound **3**: ^1^H NMR spectrum (D_2_O, 500 MHz, r.t)

**
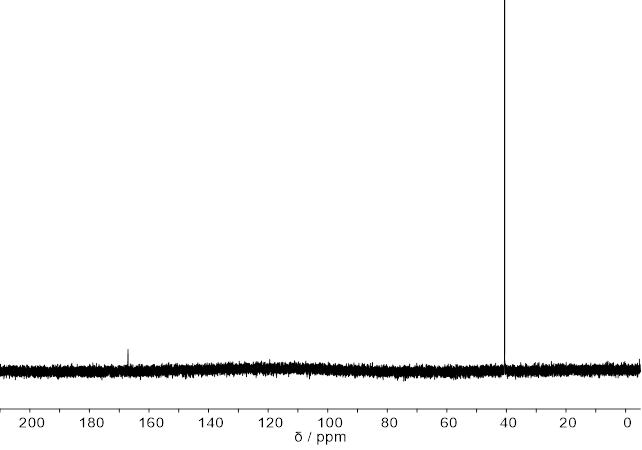
**

**Figure S41.** Compound **3**: ^13^C NMR spectrum (D_2_O, 126 MHz, r.T.)

**
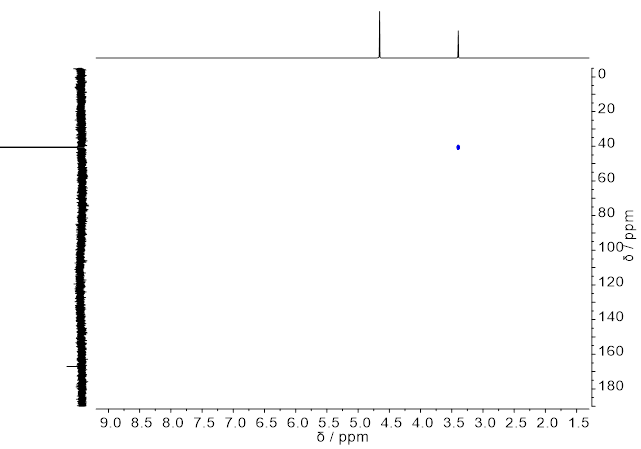
**

**Figure S42.** Compound **3**: HSQC NMR (D_2_O, 500 MHz, r.T.)

**
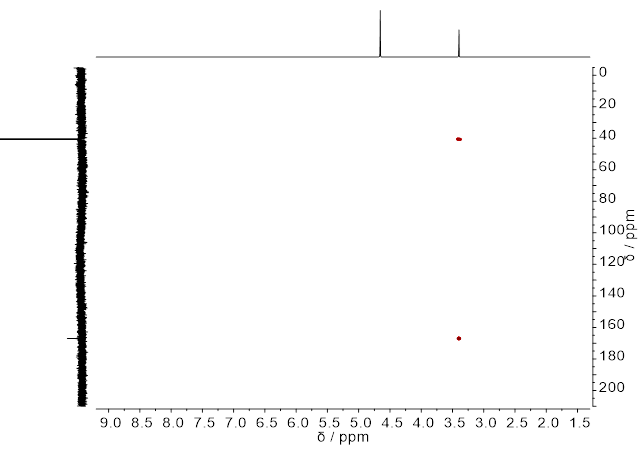
**

**Figure S43.** Compound **3**: HMBC NMR (D_2_O, 500 MHz, r.T.)


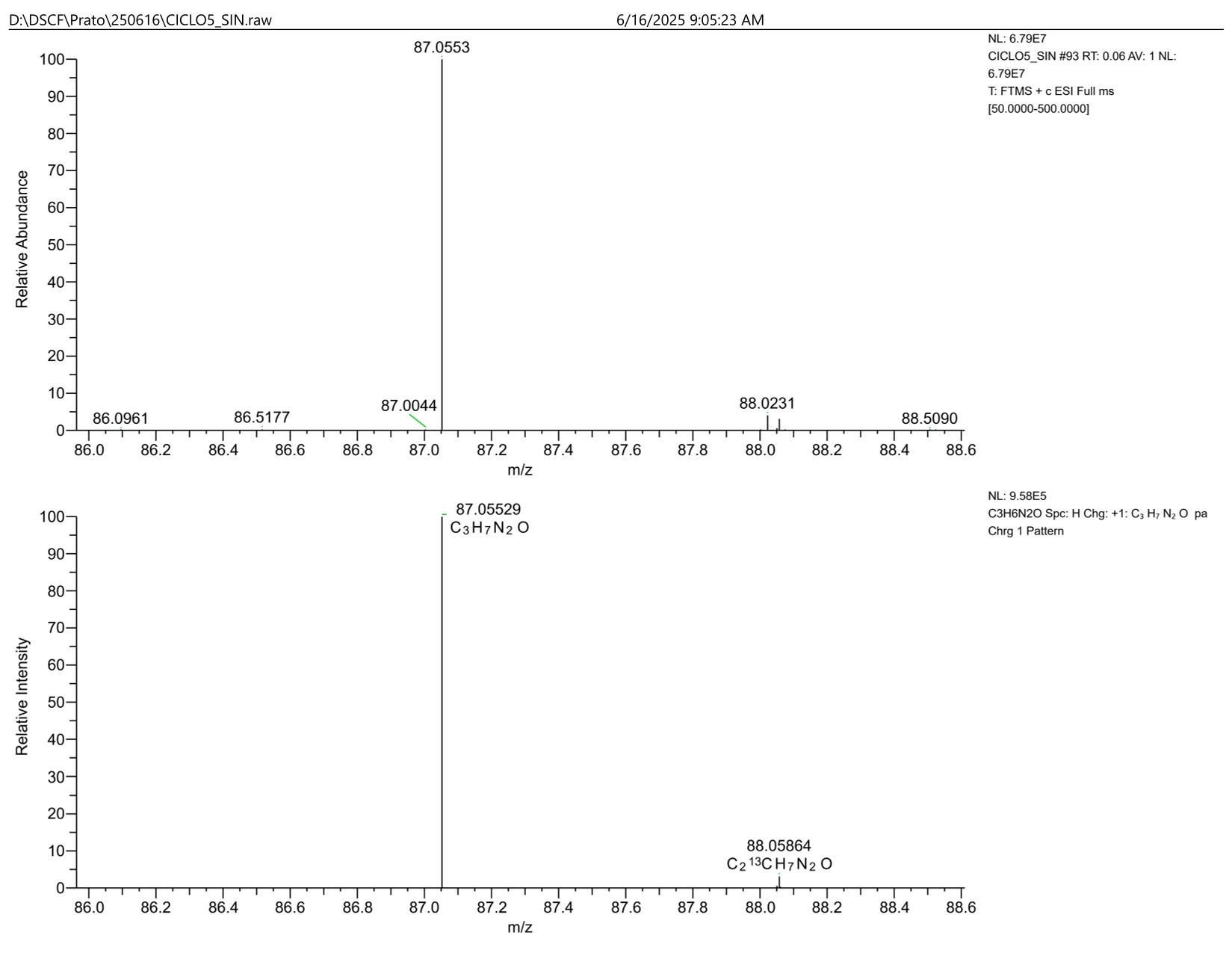


**Figure S44.** Compound **3**: HRMS, experimental spectra (top), simulated spectra (bottom).

**Figure S45.** Compound **3**: Semipreparative C8-HPLC chromatogram monitored at 215 nm.

- 1. **Compound 4**: 3-(imidazolidin-2-ylideneamino)piperidin-2-one

Prepared according to a modified literature procedure.^[5]^ 3-aminopiperidin-2-one (58 mg, 0.5 mmol, 1.6 eq.) and 2-methylthio-2-imidazoline hydroiodide (81 mg, 0.33 mmol, 1 eq.) were dissolved in 1 mL of ACN and heated in MW for 30 min at 150°C. The solvent was then removed under vacuum and the residue was purified by preparative HPLC. Compound **4** was finally obtained as a white solid (69 mg, 0.23 mmol, 45% yield).

**^1^H NMR** (500 MHz, D_2_O) δ 4.12 (dd, J = 10.3, 5.9 Hz, 1H), 3.74 (s, 4H), 3.33 (dd, J = 7.5, 4.4 Hz, 2H), 2.31 – 2.21 (m, 1H), 2.04 – 1.79 (m, 3H); **^13^C NMR** (126 MHz, D_2_O) δ 171.57, 160.20, 52.56, 42.69, 41.29, 26.69, 20.02; **HRMS** (ESI+): m/z calcd. for [C_8_H_15_N_4_O]^+^: 183.1248, found: 183.1240.


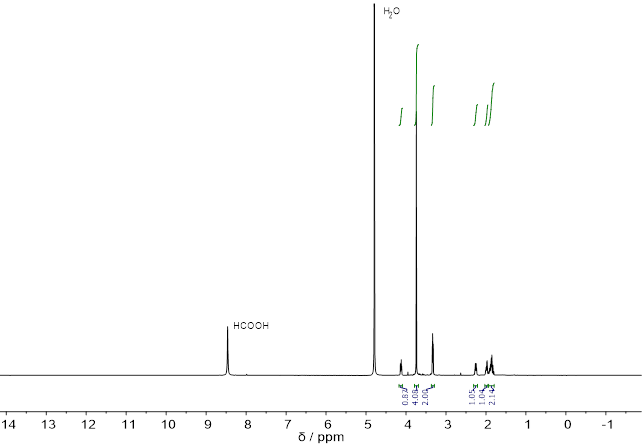


**Figure S46.** Compound **4**: ^1^H NMR spectrum (D_2_O, 500 MHz, r.T)


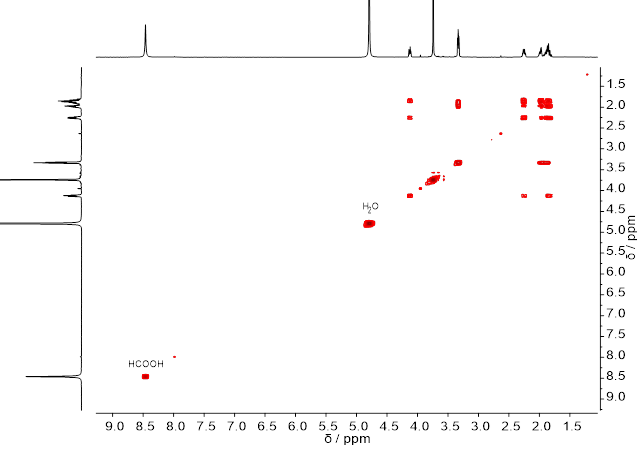


**Figure S47.** Compound **4**: COSY spectrum (D_2_O, 500 MHz, r.T)


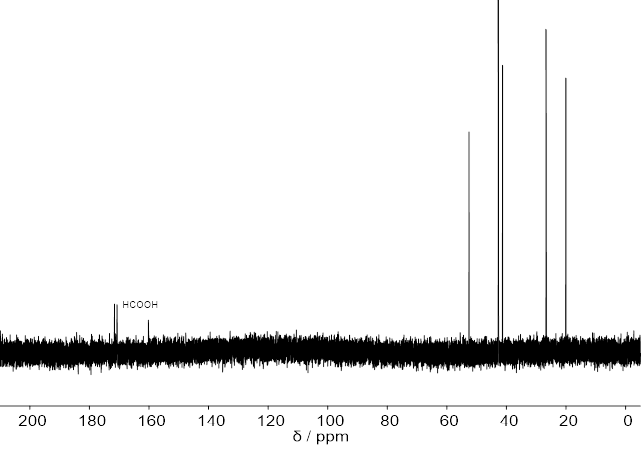


**Figure S48.** Compound **4**: ^13^C NMR spectrum (D_2_O, 126 MHz, r.T.)

**
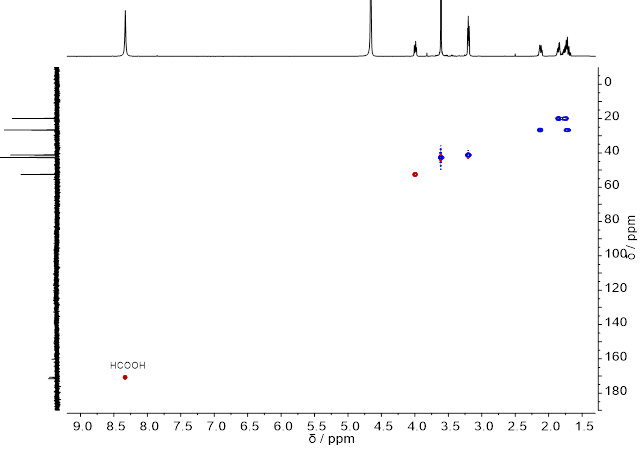
**

**Figure S49.** Compound **4**: HSQC NMR (D_2_O, 500 MHz, r.T.)


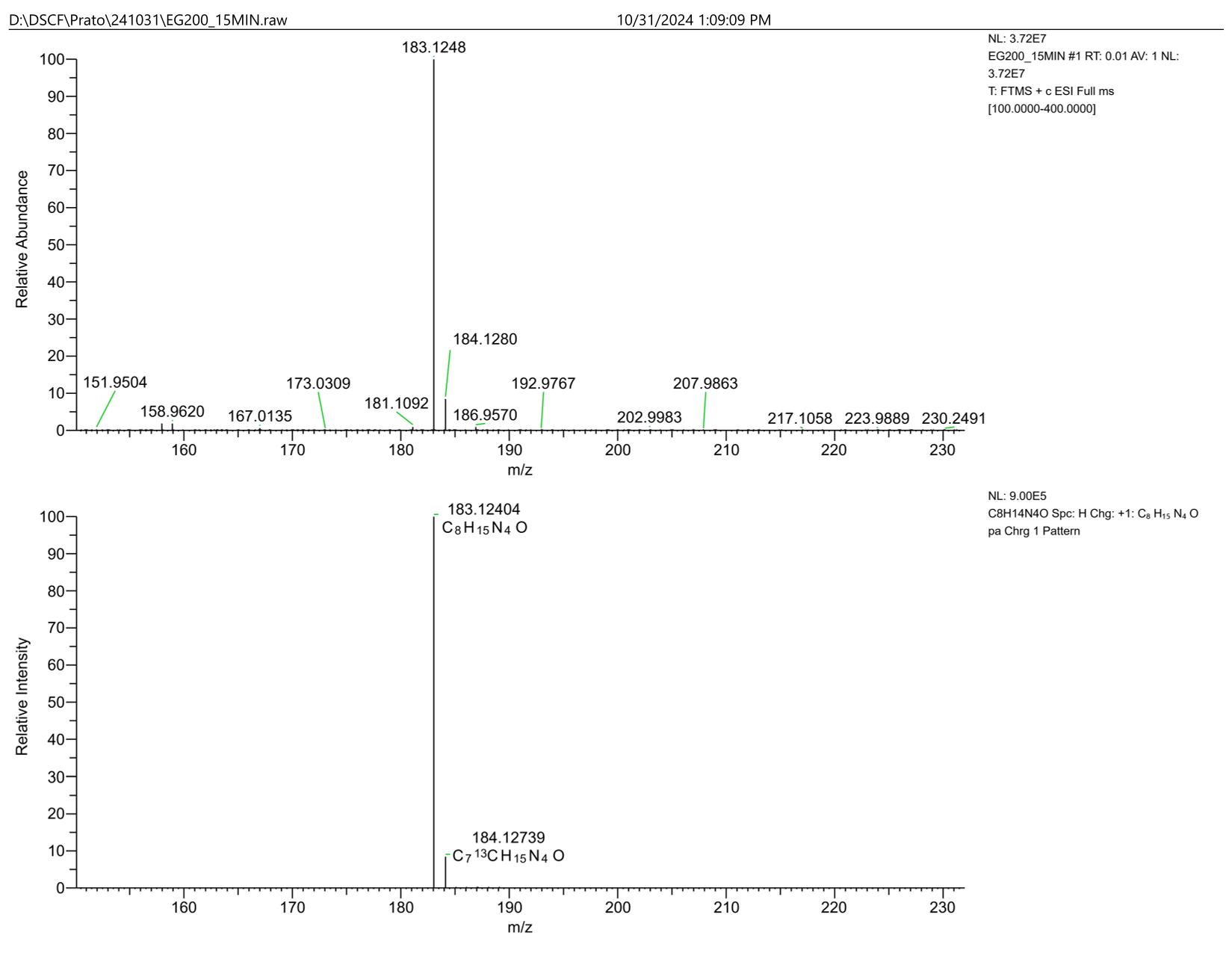


**Figure S50.** Compound **4**: HRMS, experimental spectra (top), simulated spectra (bottom)

**Figure S51.** Compound **4**: Semipreparative C8-HPLC chromatogram monitored at 215 nm.

1. Assessment of CND purity and determination of the molecular weight

**4.1 Characterization of CNDs**


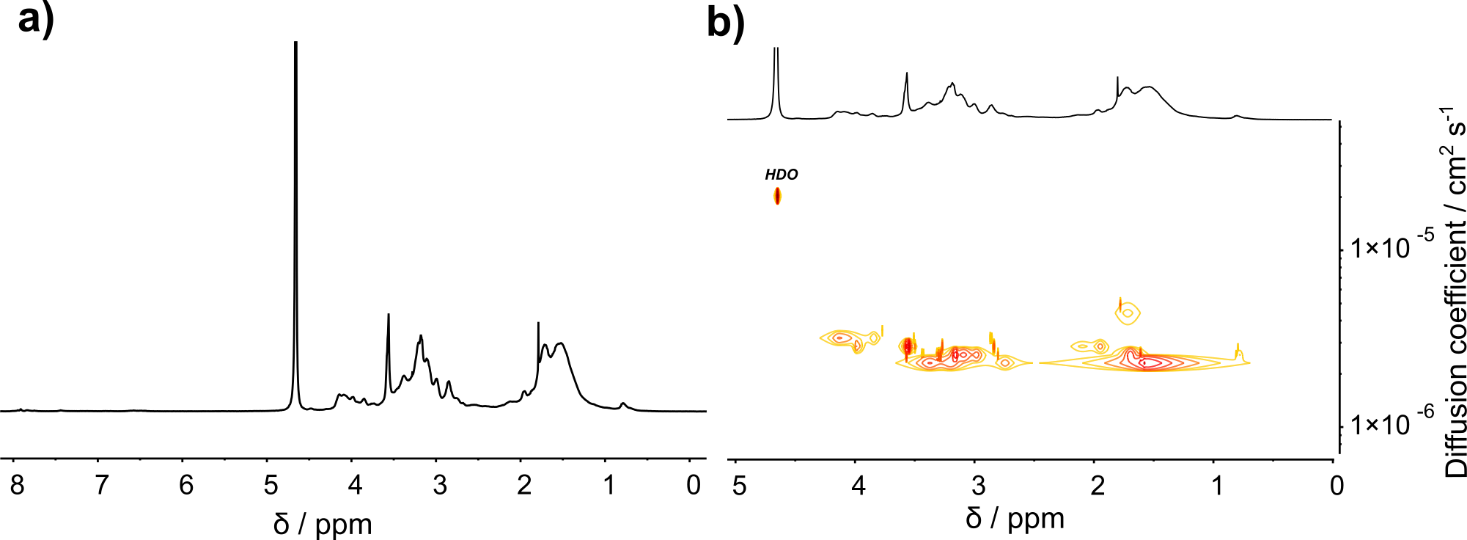


**Figure S52. a)** ^1^H NMR spectrum of CNDs recorded in deuterium oxide and referenced against solvent residual peak (δH = 4.79 ppm). **b)** DOSY spectrum of CNDs in deuterium oxide, solvent residual peak at 4.79 ppm.


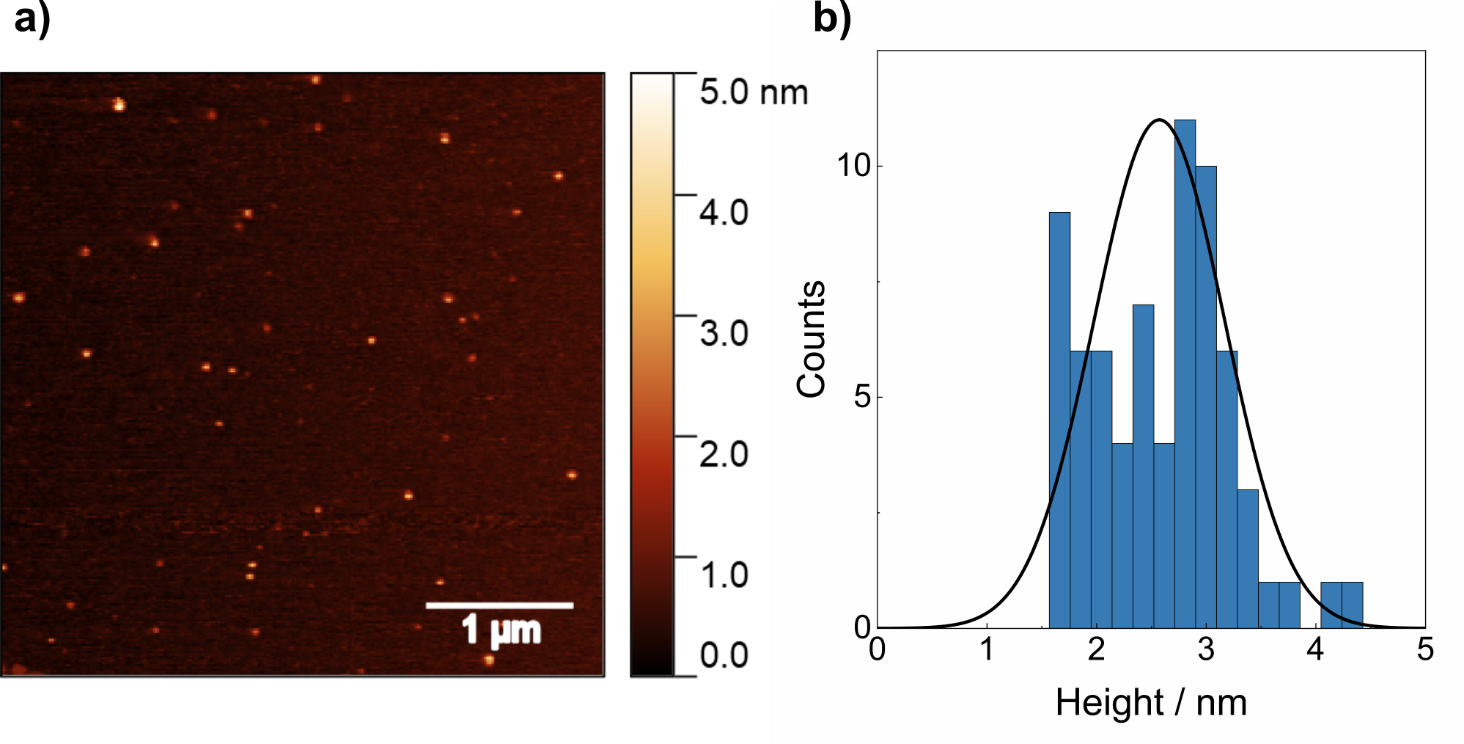


**Figure S53.** AFM characterization of purified CNDs. **a)** Micrograph of purified CNDs, color brightness indicates the relative height, from lowest (dark) to highest (bright). **b)** Size distribution of purified CNDs. The gaussian fitting curve indicates the size distribution of CNDs, which average size is 2.6 ± 0.1 nm.


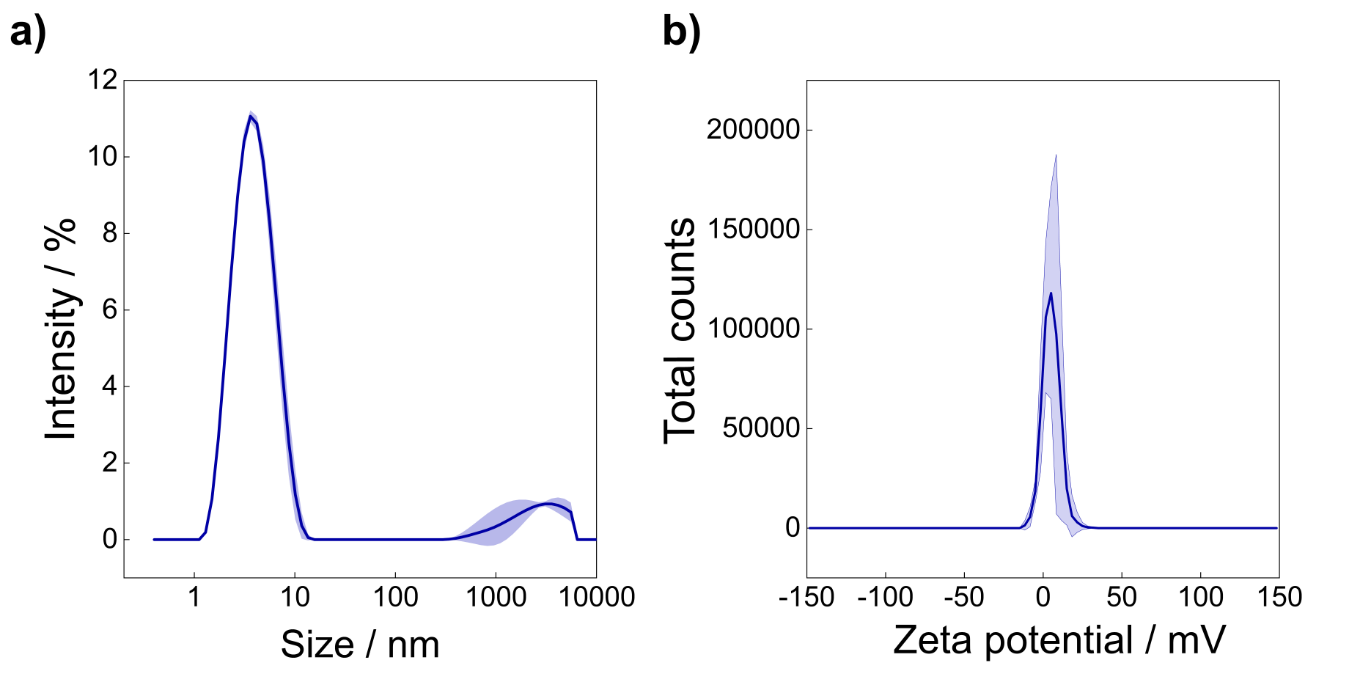


**Figure S54.** **a)** Size distribution by intensity of CNDs (5 mg mL^-1^, 0.1 M NaNO_3_ + 0.5 v/v% acetic acid, pH 2.6, 20 °C). The average size of CNDs is 3.8 ± 0.2 nm. Some aggregates are visible as a low-intensity band centred at around 1 µm. These tendency to aggregation is supported by the value of zeta potential (see **b**) which is linked to an unstable colloidal system (stable colloid ζ > +20 mV or < -20 mV). **b)** Zeta potential distribution of positively charged CNDs (5 mg mL^-1^, 0.1 M NaNO_3_ + 0.5 v/v% acetic acid, pH 2.6, 20 °C). CND zeta potential resulted ζ = +12.1 ± 2.8 mV.


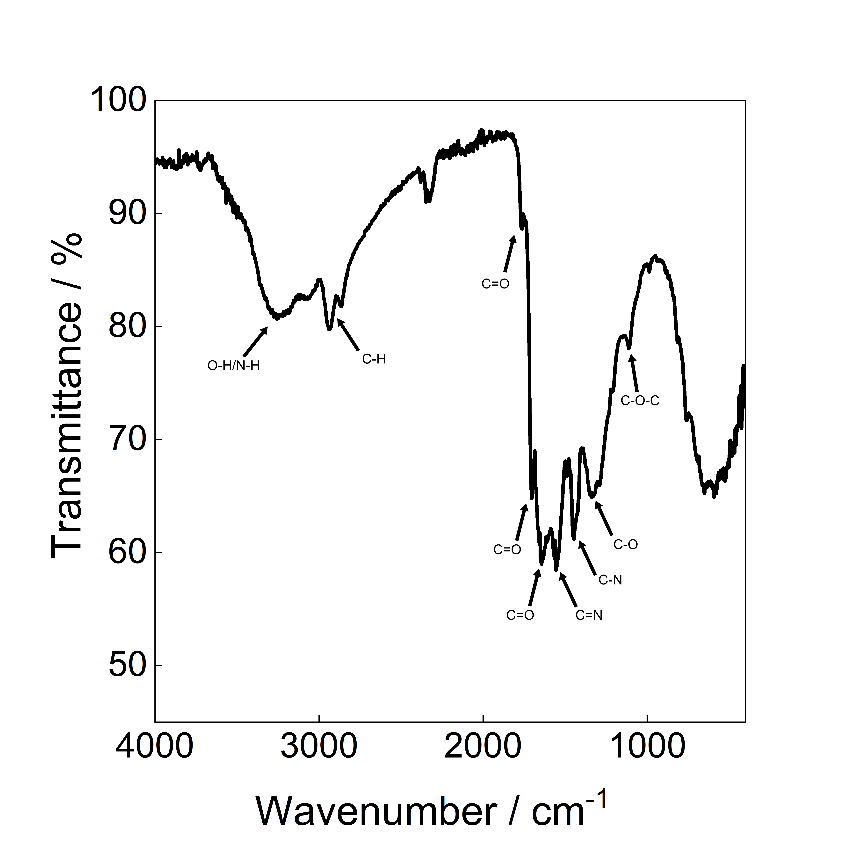


**Figure S55.** ATR-FTIR spectrum of purified CNDs. According to literature, peak assignation is as follows: C-O-C bonds (1194, 1111 cm^-1^), C-O bonds (1350, 1318 cm^-1^), C=O bonds (1655, 1704, 1767 cm^-1^), C=N (1557 cm^-1^), C-N (1492, 1437 cm^-1^), C-H bonds (2932, 2862 cm^-1^), O-H/N-H bonds (3300 cm^-1^).^[6]^


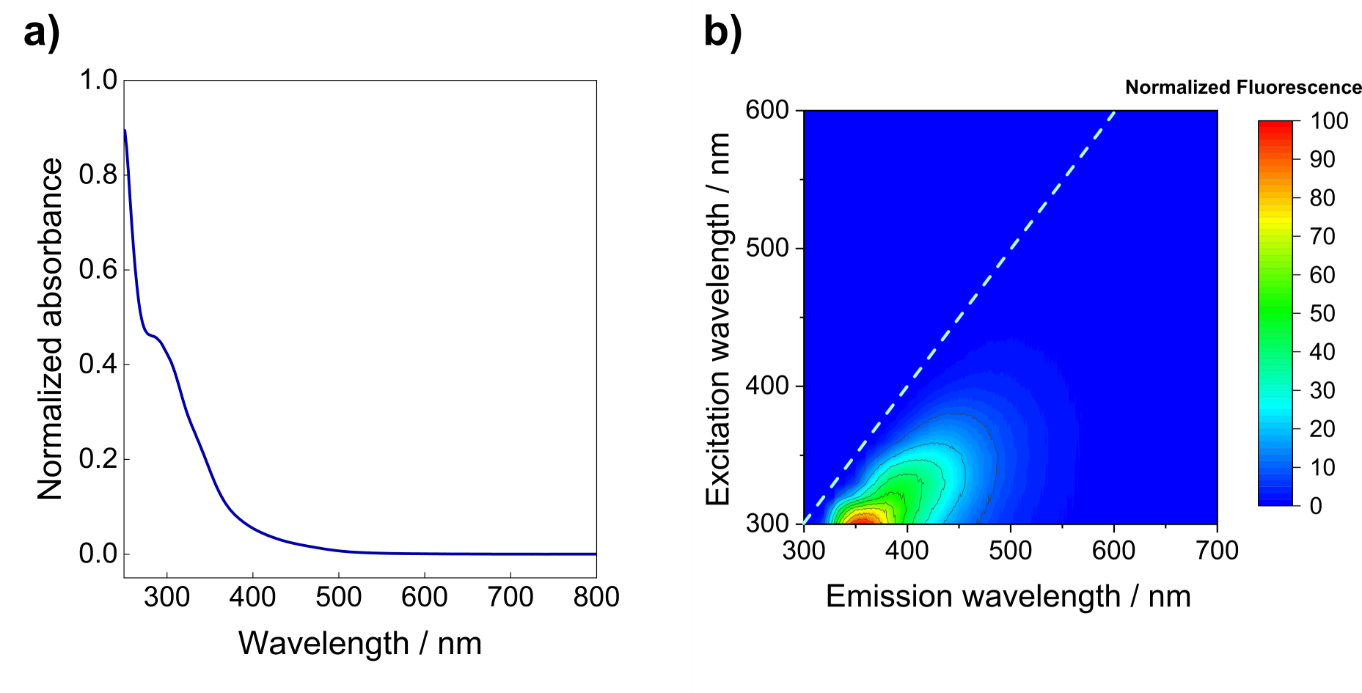


**Figure S56.** Absorption and emission properties of purified CNDs. **a)** Normalized UV-Vis spectrum of purified CNDs (1 mg mL^-1^ in Milli-Q water). **b)** Emission map of normalized fluorescence intensity of purified CNDs in Milli-Q water (λ_em_ = 356 nm corresponding to a λ_exc_ = 300 nm). The fluorescence intensities of the emission map were normalized between 0 (blue colour) and 100 (red colour). The diagonal dashed line limits the region of real values acquired during the experiments.

**4.2 Determination of CND molecular weight by MD-GPC**

We employed MD-GPC to determine the absolute molecular weight and molecular weight distribution of CNDs. This method allows molecular weight and molecular weight distribution determination without reliance on a calibration curve by combining the responses of three detectors: a light scattering detector, which directly measures the molecular weight based on the intensity of scattered light, and two concentration detectors, a refractive index (RI) detector and a UV-Vis detector. Additional detectors, such as a viscometer, can provide insights into the hydrodynamic radius (*R_H_*).

Below are reported the simplified equations (Equation 1 – 3) that allow for the determination of the absolute molecular weight from the response of the three detectors:

| $RI signal \left( mV \right)= K_{RI}\times\frac{dn}{dC}\times C$ | **Equation 1** |
| --- | --- |
| $UV signal \left( mV \right)= K_{UV}\times\frac{dA}{dC}\times C$ | **Equation 2** |
| $LS signal \left( mV \right)= K_{LS}\times MW\times\left( \frac{dn}{dC} \right)^{2}\times C$ | **Equation 3** |

In Equation 1, *K_RI_* is the constant of the RI detector, determined by calibrating the detector using a pullulan polymer standard with known molecular weight and *dn/dC*. The term *dn/dC* describes how changes in sample concentration affect the refractive index of the solution and is therefore specific to each sample/solvent system. *C* is the concentration of the sample. In Equation 2, *K_UV_* is the constant of the UV detector, determined using a bovine serum albumin standard with known molecular weight and *dA/dC*. The term *dA/dC* describes how changes in sample concentration affect the solution absorbance. In Equation 3, *K_LS_* is the constant of the light scattering detector, determined during the pullulan standard calibration.

Based on these equations, the key parameters required to calculate the absolute molecular weight and its distribution are the *dn/dC*, which is specific to the CD/solvent system, and the concentration of CND in solution. The latter could also be determined using the *dA/dC*, a parameter that can be obtained from the UV-Vis chromatogram.


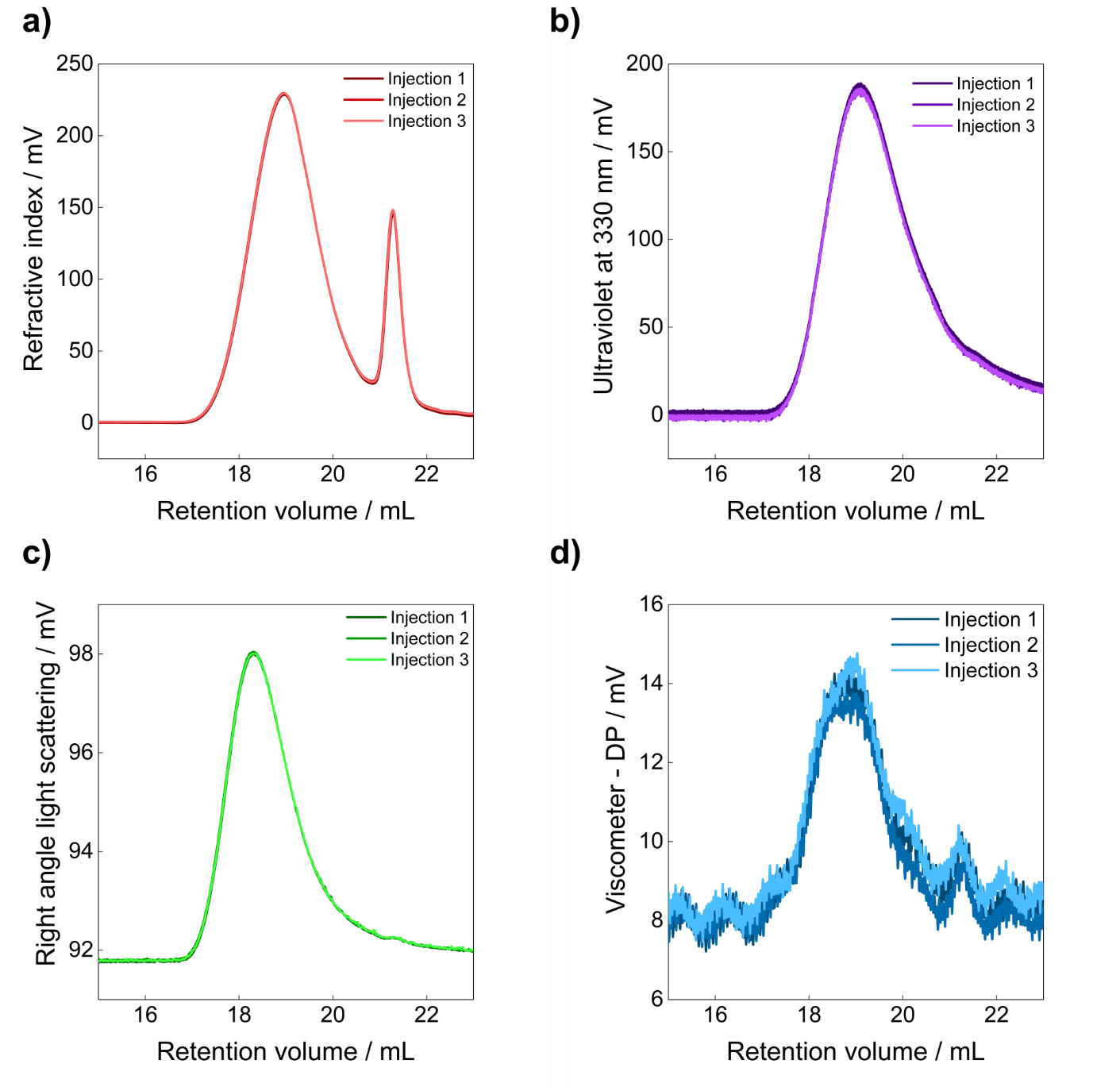


**Figure S57.** MD-GPC chromatogram overlays of three different injections of the same CND sample (concentration = 5 mg mL^-1^, 0.1 M NaNO_3_ + 0.5 v/v% acetic acid, pH 2.6 on TSKgel GMPWXL-CP (G6000 + G3000PWXL-CP) cationic columns. **a)** Refractive index elution profile (red). **b)** UV-Vis elution profile at 330 nm (purple). **c)** Light scattering elution profile (green). **d)** Viscometer (DP) elution profile (blue).

Table S1 presents a comparison of the main parameters obtained from MD-GPC analysis using the corrected concentration of CND samples, for the analyzed batches A, B, and C.

**Table S1.** Comparison table between MD-GPC average results, namely dn/dC, M_n_, M_w_, Đ, recovery and R_H_ of different batches of CNDs obtained with corrected sample concentration.

| **Batch** | ***dn/dC* (mL g^-1^)** | ***M_n_* (g mol^-1^)** | ***M_w_* (g mol^-1^)** | ***Đ*** | **Recovery (wt%)** | ***R_H_* (nm)** |
| --- | --- | --- | --- | --- | --- | --- |
| **A** | 0.195 ± 0.002 | 3,927 ± 8 | 5,272 ± 12 | 1.342 ± 0.001 | 99 | 1.41 ± 0.01 |
| **B** | 0.201 ± 0.002 | 4,815 ± 54 | 7,035 ± 85 | 1.461 ± 0.002 | 97 | 1.52 ± 0.01 |
| **C** | 0.213 ± 0.001 | 4,547 ± 34 | 6,710 ± 45 | 1.476 ± 0.002 | 96 | 1.45 ± 0.01 |
| **Average** | 0.203 ± 0.010 | 4,400 ± 458 | 6,333 ± 907 | 1.426 ± 0.073 | 97 ± 2 | 1.46 ± 0.06 |


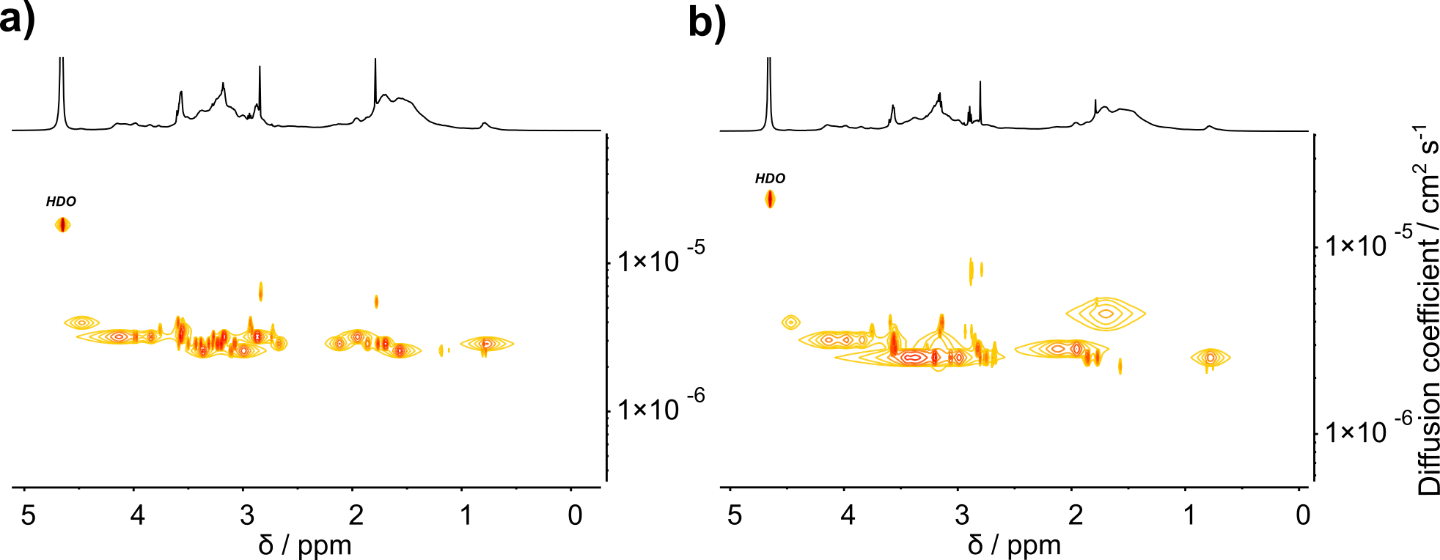


**Figure S58.** DOSY NMR spectra of batch B and C of CNDs in deuterium oxide, solvent residual peak at 4.79 ppm. **a)** batch B. **b)** batch C.

**4.3 Quantification of CND primary aliphatic amines by Kaiser Test**

Kaiser test was carried out by using a commercially available kit (Sigma Aldrich) and according to a modified procedure.^[7]^ Briefly, 1 mg of purified CNDs was weighed in a 4 mL glass vial. Then, 75 µL of a phenolic solution in ethanol (80%), 100 µL of a KCN solution in pyridine/water and 75 µL of a ninhydrin solution in ethanol (6%) were added in this specific order. The solution was left reacting at 120 °C for 10 minutes. The resulting solution was diluted with 60 V/V% ethanolic aqueous solution, and its absorption spectrum was recorded. A solution without CNDs was used as a reference blank. Aliphatic primary amines on the CNDs surface were quantified from the absorbance value at 570 nm, using a molar absorption coefficient of 15,000 M^-1^ cm^-1^ for Ruhemann’s purple complex. Experiments were repeated in triplicate.


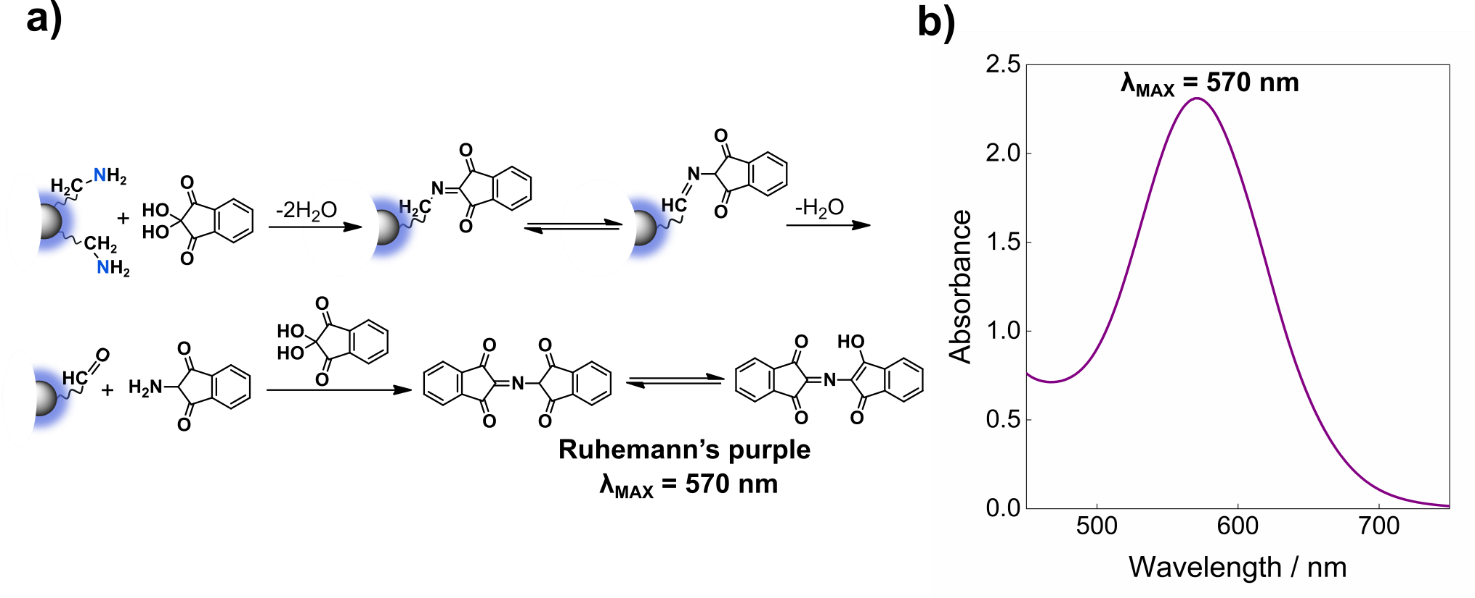


**Figure S59.** **a)** Proposed mechanism for Kaiser test reactions on CNDs.^[8]^ **b)** Absorbance maxima (λ = 570 nm) of Ruhemann’s purple complex formed after Kaiser test on CNDs performed at 120 °C.

**4.4 Quantification of CND primary amines by ^19^F NMR spectroscopy**

All the ^19^F NMR spectroscopy quantification experiments were performed by dissolving 10 mg of CNDs in 750 µL of deuterated DMSO and by adding 5 equivalents of *p-*fluorobenzaldehyde. The solution was left to react for 24 hrs at room temperature. Experiments were repeated in triplicate. All the spectra were registered at 25 °C and using trifluoro benzene as the internal standard.


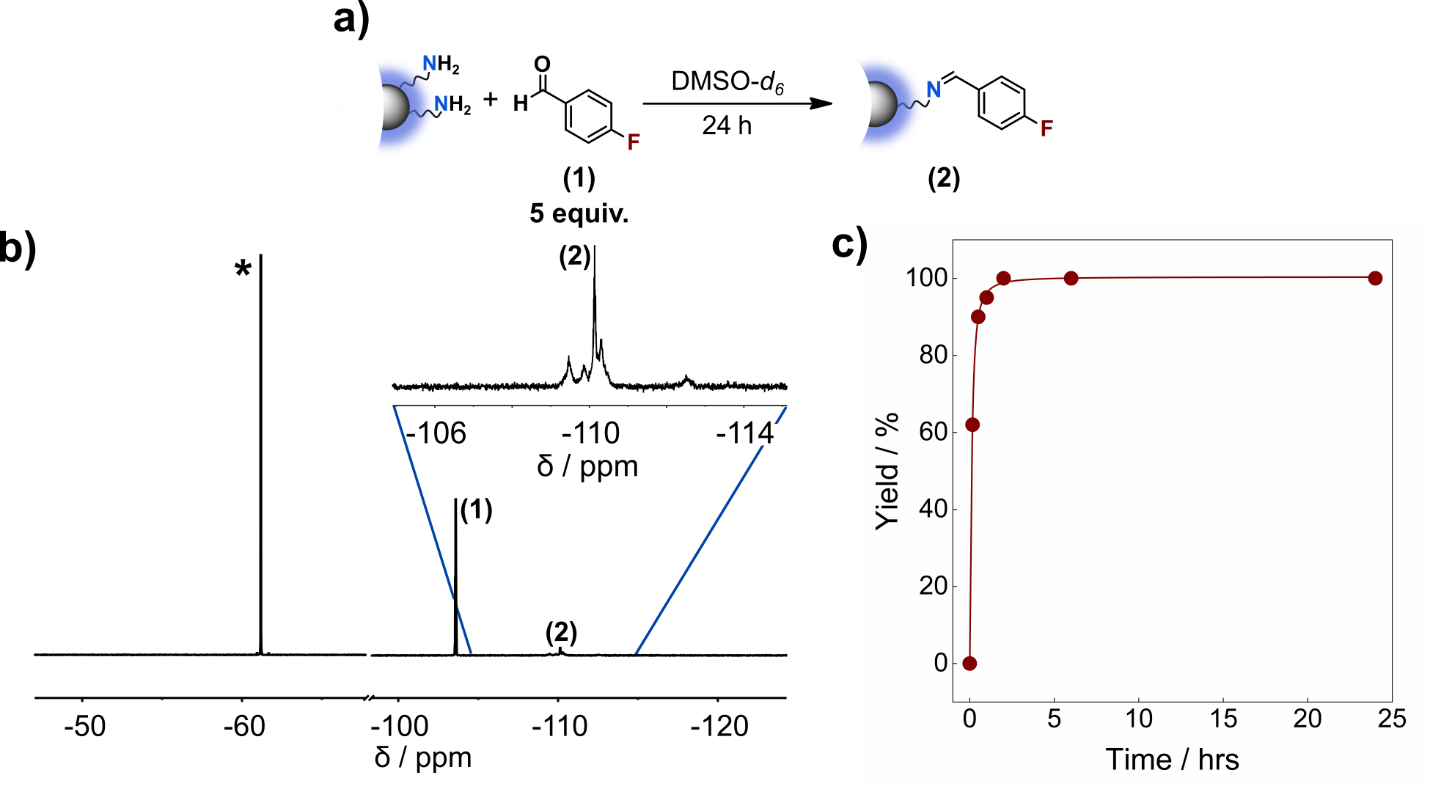


**Figure S60. a)** Reaction scheme of imine (2) formation on CND surface. **b)** ^19^F NMR spectrum in DMSO-*d6* (400 MHz, room temperature) showing successful formation of CND imine derivatives (2) (-61.18 ppm - trifluoro toluene, internal standard (*****, -103.59 ppm *p*-fluoro benzaldehyde (1), -109 – -111 ppm imines (2)). **c)** Kinetic curve of percentage imine yield formation over 24 hrs at room temperature.

1. **References**
2. R. Paul, G. W. Anderson, F. M. Callahan, *J. Org. Chem.* **1961**, *26*, 3347–3350.
3. S. V. Burov, Yu. E. Moskalenko, M. V. Leko, M. Yu. Dorosh, and E. F. Panarin, *Russ. J. Bioorg. Chem.* **2006**, *32*, 509–516.
4. I. S. Hutchinson, S. A. Matlin, A. Mete, *Tetrahedron* **2002**, *58*, 3137–3143.
5. N. Aoyagi, T. Endo, *Synthetic Communications* **2017**, *47*, 442–448.
6. B. M. Bhanage, S. Fujita, Y. Ikushima and M. Arai, *Green Chem.* **2004**, *6*, 78-80.
7. F. Arcudi, L. Đorđević, M. Prato, *Angewandte Chemie* **2016**, *128*, 2147–2152.
8. D. Iannazzo, A. Piperno, A Ferlazzo, A. Pistone, C. Milone, M. Lanza F. Cimino, A. Speciale, D. Trombetta, A. Saija, S. Galvagno, *Org Biomol Chem*, **2012**, *10*, 1025–1031.
9. G. Jarre, S. Heyer, E. Memmel, T. Meinhardt, A. Krueger, *Beilstein Journal of Organic Chemistry* **2014**, *10*, 2729–2737.
